# Targeting GLI1 Transcription Factor for Restoring Iodine Avidity with Redifferentiation in Radioactive-Iodine Refractory Thyroid Cancers

**DOI:** 10.3390/cancers14071782

**Published:** 2022-03-31

**Authors:** Ji Min Oh, Ramya Lakshmi Rajendran, Prakash Gangadaran, Chae Moon Hong, Ju Hye Jeong, Jaetae Lee, Byeong-Cheol Ahn

**Affiliations:** 1 Department of Nuclear Medicine, School of Medicine, Kyungpook National University, Daegu 41944, Korea; ojm0366@knu.ac.kr (J.M.O.); ramyag@knu.ac.kr (R.L.R.); prakashg@knu.ac.kr (P.G.); cmhong@knu.ac.kr (C.M.H.); jaetae@knu.ac.kr (J.L.); 2 BK21 FOUR KNU Convergence Educational Program of Biomedical Sciences for Creative Future Talents, Department of Biomedical Science, School of Medicine, Kyungpook National University, Daegu 41944, Korea; 3 Department of Nuclear Medicine, Kyungpook National University Hospital, Daegu 41944, Korea; zzu--@hanmail.net

**Keywords:** thyroid cancer, GLI1, redifferentiation, sodium iodide symporter, radioactive-iodine therapy

## Abstract

**Simple Summary:**

Thyroid cancers have an excellent prognosis by standard therapy of surgery followed by radioactive-iodine therapy. However, metastatic thyroid cancers do not response to radioactive-iodine therapy by losing iodine avidity. Therefore, reversing iodine avidity to metastatic thyroid cancers gives a new chance of applying radioactive-iodine therapy to the cancers. In the current study, we found that GLI1 knockdown can revert iodine non-avid thyroid cancers to iodine avid cancers by increasing expression of thyroid-specific proteins. Restoration of iodine avidity in thyroid cancers makes the cancers sensitive to radioactive-iodine therapy again. Therefore, the GLI1 can be a potential therapeutic target of radioactive-iodine resistant thyroid cancers.

**Abstract:**

Radioactive-iodine (RAI) therapy is the mainstay for patients with recurrent and metastatic thyroid cancer. However, many patients exhibit dedifferentiation characteristics along with lack of sodium iodide symporter (NIS) functionality, low expression of thyroid-specific proteins, and poor RAI uptake, leading to poor prognosis. Previous studies have demonstrated the effect of GLI family zinc finger 1 (GLI1) inhibition on tumor growth and apoptosis. In this study, we investigated the role of GLI1 in the context of redifferentiation and improvement in the efficacy of RAI therapy for thyroid cancer. We evaluated GLI1 expression in several thyroid cancer cell lines and selected TPC-1 and SW1736 cell lines showing the high expression of GLI. We performed GLI1 knockdown and evaluated the changes of thyroid-specific proteins expression, RAI uptake and I-131-mediated cytotoxicity. The effect of GANT61 (GLI1 inhibitor) on endogenous NIS expression was also assessed. Endogenous NIS expression upregulated by inhibiting GLI1, in addition, increased expression level in plasma membrane. Also, GLI1 knockdown increased expression of thyroid-specific proteins. Restoration of thyroid-specific proteins increased RAI uptake and I-131-mediated cytotoxic effect. Treatment with GANT61 also increased expression of endogenous NIS. Targeting GLI1 can be a potential strategy with redifferentiation for restoring RAI avidity in dedifferentiated thyroid cancers.

## 1. Introduction

Thyroid cancer is the most common endocrine cancer, and its incidence has shown an increasing trend over time. According to statistical analysis of GLOBOCAN 2020, estimates of cancer incidence produced by the International Agency for Research on Cancer (IARC), thyroid cancer accounted for 586,000 new incidence cases and ranked 9th in terms of global cancer burden in 2020 [1]. Papillary thyroid cancer (PTC) is the most common histological type among differentiated thyroid cancers (DTC), accounting for >80% of all thyroid cancers [2]. Most of the DTCs, including PTC, have an excellent prognosis with standard surgical thyroidectomy and radioactive-iodine (RAI) therapy, and thyroid-stimulating hormone (TSH) suppressive therapy; however, recurrence at a locoregional area and distant organs occurs in up to 20% and 10% cases, respectively [3]. RAI therapy remains the mainstay of therapy for surgically non-removable recurrences; however, two-thirds of the lesions show no or minimal uptake of RAI [3,4]. Eventually, RAI therapy shows no clinically beneficial effects due to dedifferentiation of DTC, a condition referred to as RAI-refractory DTC. Many studies have revealed the association of several aberrant signaling pathways (including MAPK and PI3K/AKT) and epigenetic alterations (such as DNA methylation and histone modification) with dedifferentiation of thyroid cancers [5]. Thus, identification of novel redifferentiation strategies based on molecular mechanisms underlying dedifferentiation is a key imperative.

Hedgehog signaling pathway, which is activated by three ligands (Sonic, Indian, Desert), plays an important role in embryonic development (both vertebrates and invertebrates), tissue regeneration, and stem cell renewal [6,7]. A study demonstrated the association between hedgehog signaling pathway and tumorigenesis by identifying the *PATCHED* mutation, which is the receptor of the hedgehog ligand, in several types of cancers [8]. Subsequently, several reports verified that activation of hedgehog signaling pathway may be linked to tumorigenesis without mutation [9,10,11,12]. Of note, the GLI family zinc finger 1 (*GLI1*), which is a known glioma-associated oncogene, is a crucial transcription factor in the hedgehog signaling pathway. It can activate various target genes that are involved in hedgehog signaling pathway feedback (e.g., *GLI1*, *PTCH1*), genetic instability (e.g., p53), proliferation (e.g., *Cyclin-D2*, *MYC*, *HES1*), apoptosis (e.g., *BCL-2*), epithelial-to-mesenchymal transition (e.g., *SNAIL*), stem cell self-renewal (e.g., *NANOG*, *SOX2*), and angiogenesis (e.g., *VEGF*, *ANG1/2*) [13,14]. Parascandolo et al. demonstrated the association between GLI1 expression and various types of thyroid cancers [15]. They found that all thyroid tumors, regardless of their histological type, were positive for GLI1 protein, while all normal thyroid tissues were negative. Moreover, Lee et al. reported an association of the expression levels of GLI1 and GLI2 transcription factors with extrathyroidal extension and lymph node metastasis in patients with PTC [16]. These findings indicate that the hedgehog signaling pathways, especially GLI1, are closely associated with thyroid cancers [15]. In a previous study, overexpression of la ribonucleoprotein domain family member (LARP) 7 was shown to upregulate sodium iodide symporter (NIS; SLC5A5; solute carrier family 5, member 5) expression and RAI uptake in PTC [17]. In addition, NIS expression was inhibited by stimulating the sonic hedgehog (SHH) signaling pathway. Even though this study focused on LARP7 for re-induction of NIS, the findings suggested that the hedgehog signaling pathway may influence the increase in factors related to RAI uptake. However, the direct link between GLI1 and RAI uptake in thyroid cancers is not clear.

Here, we investigated the role of GLI1 in the context of redifferentiation of RAI-refractory thyroid cancer. Furthermore, we also evaluated the possibility of RAI therapy following restoration of the thyroid-specific proteins and RAI uptake.

## 2. Materials and Methods

### 2.1. Cell Culture

Papillary thyroid cancer cell line (TPC-1) and anaplastic thyroid cancer cell line (SW1736) were gifts provided by Dr. Minho Shong (School of Medicine, Chungnam National University, Daejeon, Korea). Two anaplastic thyroid cancer cell lines (BHT101 and CAL62) and a papillary thyroid cancer cell line (BCPAP) were purchased from Deutsche Sammlung von Mikroorganismen and Zellkulturen (DSMZ, Braunschweig, Germany). K1, a papillary thyroid cancer cell line, was purchased from Sigma-Aldrich (St. Louis, MI, USA). The BCPAP, CAL62, and TPC-1 cells were maintained in Dulbecco’s modified Eagle’s (DMEM high glucose) medium (HyClone, Logan, UT, USA) supplemented with 10% fetal bovine serum (FBS; Gibco, Grand Island, NY, USA) and 1% penicillin–streptomycin (HyClone). The BHT101 cells were cultured in DMEM high glucose medium supplemented with 20% FBS and 1% penicillin–streptomycin. The SW1736 cells were cultured in Roswell Park Memorial Institute (RPMI)-1640 medium (HyClone) supplemented with 10% FBS and 1% penicillin–streptomycin_._ The K1 cells were maintained in Dulbecco’s modified Eagle’s medium (DMEM)/Ham’s F12 50/50 medium (Corning, Grand Island, NY, USA) supplemented with 10% FBS and 1% penicillin-streptomycin. All cell lines were maintained in a humidified incubator at 37 °C with 5% CO_2_.

### 2.2. siRNA Transfection

Scrambled siRNA (Catalog ID: D-001810-10-05), GLI1 siRNA (Catalog ID: L-003896-00-0005) and NIS siRNA (Catalog ID: L-007593-00-0005) were purchased from Dharmacon (Lafayette, CO, USA). Before starting the experiment, siRNA was dissolved in siRNA buffer (Dharmacon) and prepared 250 µM stock. 7 × 10^4^ cells/500 µL were seeded in 24-well plate in a humidified incubator at 37 °C with 5% CO_2_ overnight_._ On the next day, the medium was replaced with Opti-MEM medium (Gibco, Waltham, MA, USA) and incubated for 1 h. The siRNA (final concentration: 40 nM) was diluted in appropriate DharmaFECT transfection reagent (Dharmacon) and exposed to cells for 48 h.

### 2.3. Chemical

GANT61, a GLI1 inhibitor, was purchased from Selleckchem (Houston, TA, USA). A 1 mM stock solution was prepared by dissolving GANT61 in absolute ethanol and stored at −20 °C.

### 2.4. Cell Viability Assay

TPC-1 cells (5 × 10^3^ cells/100 µL) were seeded in 96-well plates containing medium supplemented with 2.5% FBS and 1% penicillin-streptomycin, and then incubated overnight in a humidified incubator at 37 °C with 5% CO_2_. Following incubation, various concentrations of GANT61 were applied to cells for 24, 48, and 72 h, respectively. The cell viability by treating GANT61 against TPC-1 cells was determined using cell counting kit-8 assay (CCK-8 reagent; Dojindo, Kumamoto, Kumamoto, Japan). Absorbance at 450 nm was measured on a microplate reader (Model 680, Bio-Rad Laboratories, Hercules, CA, USA) and the cell viability was calculated relative to control and represented as percentage (%).

### 2.5. Protein Extraction

Cells were washed in chilled phosphate buffered saline (PBS). After removal of the PBS, the cells were exposed to Trypsin-EDTA (0.25% Trypsin with 2.25 mM EDTA) for 3 min in a humidified incubator at 37 °C with 5% CO_2_ to detach from culture plates and added the complete culture medium to inactivate Trypsin. Next, cells were centrifuged at 1500× *g* for 3 min and removed the supernatant. The collected cell pellets were lysed using radioimmunoprecipitation assay (RIPA) buffer (Thermo Fisher Scientific, Rockford, IL, USA) containing a protease and phosphatase inhibitor cocktail kit (Thermo Fisher Scientific) to isolate total protein. Lysed cells were briefly vortexed three times at 10-min intervals and centrifuged at 13,000× *g* for 20 min at 4 °C. After centrifugation, the supernatant containing total proteins was isolated. In addition, membrane proteins were extracted using Cell Surface Protein Biotinylation and Isolation Kit (Thermo Fisher Scientific) according to the manufacturer’s protocol. Protein samples were quantified using the bicinchoninic acid (BCA) protein assay kit (Thermo Fisher Scientific).

### 2.6. Western Blot Analysis

The extracted proteins diluted the 4× Laemmli sample buffer (Catalog number: #161-0747, Bio-Rad Laboratories) including 2-mercaptoethanol (Catalog number: #161-0710, Bio-Rad Laboratories) with a final concentration of 355 mM. To denature, the protein samples were boiled in a heating block for 5 min at 95 °C and keep on ice before use. Equal amounts were electrophoresed on 10% sodium dodecyl sulfate polyacrylamide gels and transferred onto polyvinylidene difluoride (PVDF) membranes (Millipore, Burlington, MA, USA). Total proteins of 20 μg extracted from whole cell lysates were loaded. In case of membrane proteins, which extracted using Cell Surface Protein Biotinylation and Isolation Kit (Thermo Fisher Scientific), were loaded 60 μg proteins. Membranes were blocked with 3% bovine serum albumin (BSA; GenDEPOT, Katy, TA, USA) prepared in Tris-buffered saline containing 0.05% Tween-20 (TBS-T; BIOSESANG, Seongnam-si, Gyeonggi-do, Korea) for 2 h and then probed overnight with primary antibodies diluted in 0.5% BSA at 4 °C. After three washes with TBS-T, the membranes were probed with horseradish peroxidase (HRP)-conjugated secondary antibodies diluted in 0.5% BSA for 1 h at room temperature (RT) and then washed three times with TBS-T. Next, membranes were exposed in an Amersham™ ECL Select™ Western Blotting Detection Reagent (Cytiva, Marlborough, MA, USA) and detected using Fusion FX chemiluminescence analyzer system (Vilber Lourmat, Marne-la-Valle’e, France) as per the manufacturer’s instructions. Band intensities were quantified using a chemiluminescence analyzer system. The list of used primary antibodies were represented in Scheme S1. Anti-mouse IgG, HRP-linked antibody (Catalog Number: #7076; Cell Signaling, Danvers, MA, USA) and anti-rabbit IgG, HRP-linked antibody (Catalog Number: #7074; Cell Signaling) as secondary antibodies were used and details for dilution ratio represented in Scheme S1. Blot images were cropped and prepared using PowerPoint (Microsoft, Redmond, WA, USA); the contrast was adjusted using Fusion FX chemiluminescence analyzer system or PowerPoint, if necessary, for better visualization.

### 2.7. Immunofluorescence

The cells (6 × 10^4^ cells/500 µL) were seeded and incubated for 24 h in a 4-well chamber at 37 °C in a 5% CO_2_ humidified atmosphere. After changing the culture medium to 500 µL Opti-MEM, the cells were treated with scrambled siRNA, GLI1 siRNA or NIS siRNA for 48 h. Subsequently, cells were washed three times with PBS and fixed using 4% paraformaldehyde (BIOSESANG) dissolved in 1X PBS for 10 min at RT. Thereafter, cells were rinsed three times with PBS and underwent the blocking step by using 3% BSA in PBS with Tween-20 (PBS-T) of 0.05% for 1 h. Cells were probed with both NIS antibody conjugated with AF488 (working dilution 1:250, wavelength excitation at 493 nm; emission at 517 nm, catalog number: NBP1-70342AF488, Novus Biologicals, Centennial, CO, USA) and sodium potassium ATPase (Na^+^/K^+^ ATPase) antibody conjugated with AF647 (working dilution 1:250, wavelength excitation at 653 nm; emission at 668 nm, catalog number: ab198367, Abcam, Cambridge, United Kingdom) at 4 °C overnight. On the next day, the cells were washed with PBS three times and Hoechst 33342 (working dilution 1:2000 in 1X PBS, wavelength excitation at 348 nm; emission at 455 nm, catalog number: H3570, Thermo Fisher Scientific) added and incubated for 10 min at RT. Then, the cells were washed three times and coverslips were mounted onto slides with VECTASHIELD antifade mounting medium (catalog number: H-1000, Vector Laboratories, Burlingame, CA, USA) and observed using confocal laser microscopy (LSM 5 exciter; Zeiss, Oberkochen, Germany).

### 2.8. I-125 Uptake Assay

Both TPC-1 and SW1736 cells (7 × 10^4^/500 µL) were seeded using each DMEM high glucose medium and RPMI1640 medium supplemented with 10% FBS and 1% penicillin-streptomycin in 24-well plates. After 24 h incubation, medium was replaced to Opti-MEM medium and treated both scrambled siRNA and GLI1 siRNA, and then incubated for 48 h in a CO_2_ incubator at 37 °C with 5% CO_2_. Following incubation, the cells were washed with pre-warmed Hank’s balanced salt solution (HBSS; HyClone) containing 0.5% BSA (bHBSS). Next, 37 kBq carrier-free I-125 (Perkin Elmer Life Science, Waltham, MA, USA) and 100 μM sodium iodide (NaI, specific activity of 740 MBq/mM, Sigma) were added and the cells incubated for 30 min in a humidified incubator at 37 °C with 5% CO_2_. To prevent I-125 uptake, the cells were treated with 50 μM potassium perchlorate (KClO_4_, Sigma), which is a competitive inhibitor of iodide transport, for 30 min before adding I-125. After incubation, the cells were washed twice with chilled bHBSS and lysed with 500 μL of RIPA buffer. Radioactivity was measured using a Cobra II gamma counter (Canberra Packard, Mississauga, ON, Canada). Uptake values were normalized with the protein concentration, as determined by BCA protein assay kit. Results are presented as count per minute (cpm)/μg.

### 2.9. I-131 Clonogenic Assay

TPC-1 cells (2 × 10^5^ cells/2 mL) were seeded using DMEM high glucose medium supplemented with 10% FBS and 1% penicillin-streptomycin in a 6-well plate and incubated for 24 h in a humidified incubator at 37 °C with 5% CO_2_ overnight. On the next day, medium was replaced to Opti-MEM medium and treated both scrambled siRNA and GLI1 siRNA, and was then incubated for 48 h. After incubation, the medium was aspirated, and the cells were rinsed twice with bHBSS. Thereafter, the cells were incubated for 7 h at 37 °C with or without 50 μCi/mL I-131 (KIRAMS, Seoul, Korea) supplemented with 30 μM NaI. Next, the cells were washed twice by bHBSS, trypsinized, counted, and re-seeded into new 6-well plates at a density of 1 × 10^3^ cells/2 mL in each well. Cells were then incubated in a humidified incubator at 37 °C with 5% CO_2_ for 10 days to enable colony formation. On day 10, cells were washed twice by PBS and the colonies were fixed with fixation buffer containing 1:7 acetic acid-methanol. The fixed colonies were stained with 0.05% crystal violet for 1 h and immersed in tap water to rinse off the extra crystal violet. Colonies containing over 50 cells in each treatment group were counted. Results are presented as survival fraction (%), which were calculated as plating efficacy (PE) of the treated sample divided by PE of the scrambled siRNA without I-131.

### 2.10. Statistical Analysis

All data are expressed as mean ± standard deviation. Between-group differences were assessed using Student’s *t*-test. Statistical analyses were performed using GraphPad Prism 5 software version 5.01 (GraphPad Software, Inc., La Jolla, CA, USA). *p* values < 0.05 were considered indicative of statistical significance.

## 3. Results

### 3.1. Comparison of Factors of Hedgehog Signaling Pathway including GLI1 in Thyroid Cancer-Derived Cell Lines

**Figure 1:**
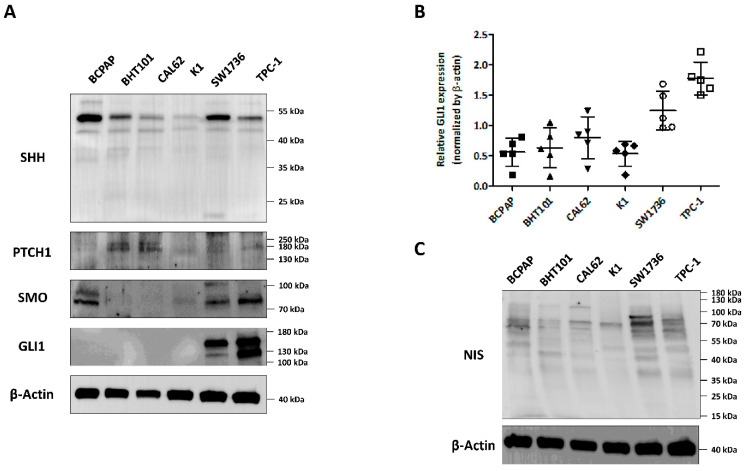
Investigation of hedgehog signaling pathway in thyroid cancer-derived cell lines. Three papillary thyroid cancer cell lines (BCPAP, K1, TPC-1) and three anaplastic thyroid cancer cell lines (BHT101, CAL62, SW1736) were subjected to western blot analysis. (**A**) Evaluation of the hedgehog signaling pathway. (**B**) Quantitative analysis of GLI1 expression in several thyroid cancer cell lines. ß-actin was used as an internal control. Mean ± standard deviation (SD) values from three independent experiments are presented. (**C**) Confirmation of NIS protein expression level in thyroid cancer-derived cell lines. ß-actin was used as an internal control. Raw data is presented in Figures S2–S4.

First, we compared the expression level of factors concerning hedgehog signaling pathway in 6 thyroid cancer cell lines (PTC cell lines: BCPAP, K1, and TPC-1; anaplastic thyroid cancer (ATC) cell lines: BHT101, CAL62 and SW1736) (Figure 1A). SHH protein (47 kDa: SHH precursor, 19 kDa: amino-terminal peptide) was expressed in all 6 cell lines, especially SW1736 showed highest expression at 19 kDa amino-terminal peptide. Patched-1 (PTCH1) receptor, which activated by binding SHH, marginally expressed all thyroid cancer cell lines, especially more expressed in BHT101 and CAL62 cells. In addition, smoothened (SMO) receptor, which is controlled by PTCH receptor, was highly expressed in BCPAP, TPC-1 and SW1736 while other cell lines were weakly expressed. Next, expression of GLI1, which was activated by the upstream components of the hedgehog signaling pathway, was strongly expressed in TPC-1 and SW1736 (Figure 1B), but there was no difference in GLI1 expression between PTC and ATC cells. These results showed that components of the hedgehog signaling pathway were activated regardless of the type of thyroid cancer cells. Moreover, NIS expression was variable in thyroid cancer cells regardless of their characteristics (Figure 1C). Based on these results, TPC-1 and SW1736 thyroid cancer cells, which were highly expressed in the GLI1, were selected for subsequent experiments.

### 3.2. Robust Effect of GLI1 Knockdown on Upregulation of Endogenous NIS Expression and Its Localization in Thyroid Cancer Cells

Aberrant silencing of NIS expression is a major mechanism of RAI refractoriness in thyroid cancers. Therefore, recuperation of NIS expression is one of the most important factors for RAI avidity in thyroid cancers. At first, we investigated the appropriate concentrations of GLI1 siRNA for NIS expression in TPC-1 cells. GLI1 knockdown led to a dose-dependent increase the NIS expression in TPC-1 cells (Supplemental Materials Figure S1A). Therefore, 40 nM siRNA was selected for a further experiment.

**Figure 2:**
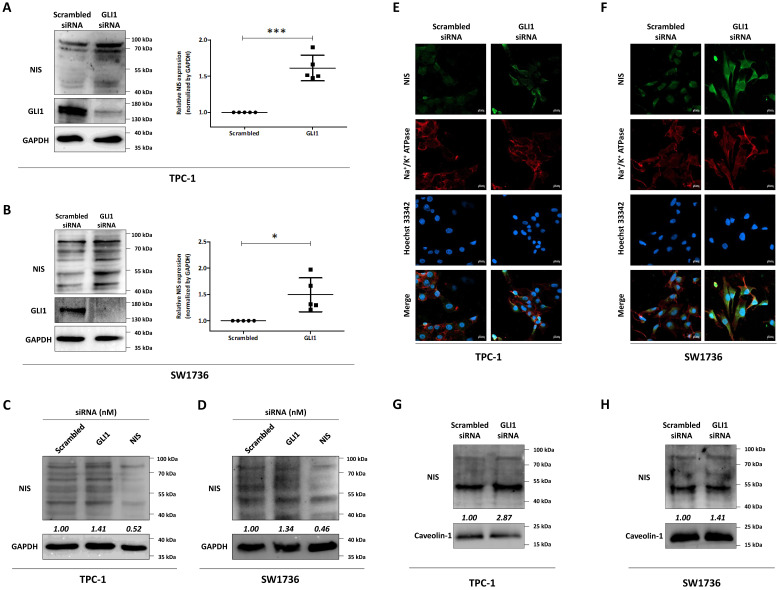
Changes in endogenous NIS expression and its localization in GLI1-inhibited thyroid cancer cells. Both TPC-1 and SW1736 cells were treated with scrambled siRNA, GLI1 siRNA or NIS siRNA for 48 h. (**A**) Changes in NIS and GLI1 expression induced by GLI1 siRNA treatment in TPC-1 cells. GAPDH was used as an internal control. Mean ± SD values from five independent experiments are presented. *** *p* < 0.001 (Student’s *t*-test). (**B**) Evaluation of NIS expression via GLI1 knockdown in SW1736 cells. Mean ± SD values from five independent experiments are presented. * *p* < 0.05 (Student’s *t*-test). Western blot analysis for NIS protein with scrambled siRNA, GLI1 siRNA or NIS siRNA treatment to TPC-1 cells (**C**) and SW1736 cells (**D**). Immunofluorescence images showing localization of endogenous NIS expression in TPC-1 cells (**E**) and SW1736 cells (**F**). Scale bar: 20 µm. Expression of endogenous NIS protein in plasma membrane fraction by GLI1 knockdown in TPC-1 cells (**G**) and SW1736 cells (**H**). Caveolin-1 were used as loading controls for plasma membrane proteins. Raw data is presented in Figures S5–S10.

Next, we sought to observe an inverse relationship between the expression of GLI1 and NIS proteins in thyroid cancer cells (Figure 2). As shown, western blot image in Figure 2A,B, endogenous NIS protein expression was upregulated by inhibiting GLI1. In addition, we performed the quantitative analysis with bands between 70 to 100 kDa, which is the size of NIS protein with full glycosylation and showed a 1.61-fold increase of endogenous NIS protein expression in TPC-1 cells (Figure 2A). SW1736 cells also showed a 1.49-fold increase in endogenous NIS protein expression by inhibiting GLI1 as a result of quantification of NIS proteins between 70 to 100 kDa (Figure 2B). Moreover, we used NIS siRNA to verify the specificity of NIS antibody detection in both cell lines and confirmed the specificity of the NIS antibody (Figure 2C,D and Figure S1B,C).

Another important determinant of NIS functionality is the membrane targeting of NIS (translocation of NIS from cytoplasm to membrane). Therefore, we examined the cellular localization of endogenous NIS by GLI1 knockdown in both thyroid cancer cells. In scrambled siRNA, endogenous NIS expression was low and was mostly expressed in the cytoplasm rather than in the membrane in both thyroid cancer cells. However, knockdown of GLI1 causes an increase of endogenous NIS protein expression and induced its localization to membrane (Figure 2E,F). Western blot analysis with plasma membrane fraction also showed a high level of NIS protein in the plasma membrane of thyroid cancer cells (Figure 2G,H). These results demonstrated that GLI1 knockdown could promote an increase in endogenous NIS expression overall and trafficking of the protein to the plasma membrane.

### 3.3. The Change of Thyroid-Specific Proteins and Transcription Factors via GLI1 Knockdown for Redifferentiation

**Figure 3:**
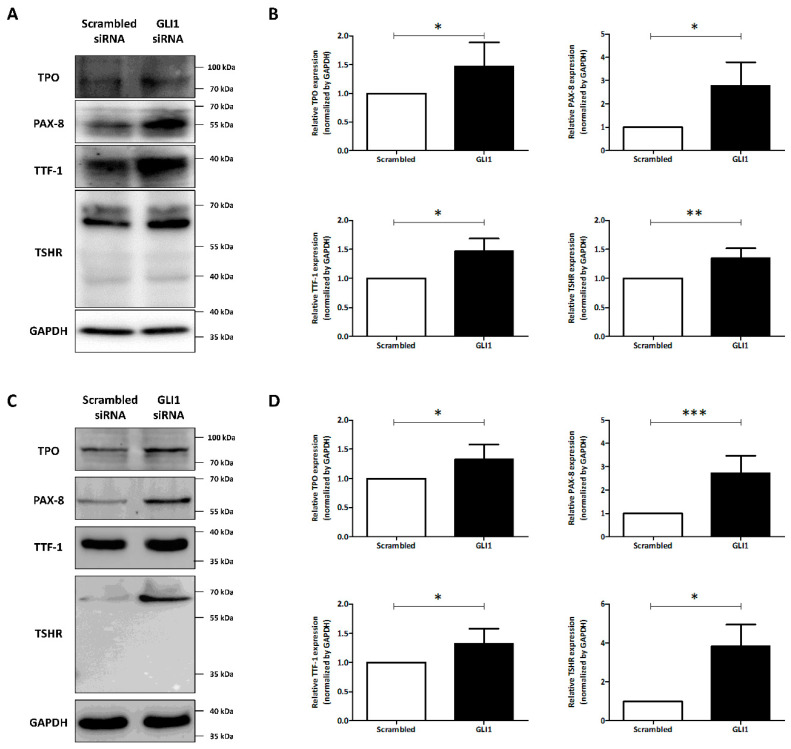
Evaluation of the expression of thyroid-specific proteins and transcription factors in thyroid cancer-derived cells with GLI1 knockdown. Both TPC-1 and SW1736 cells were treated with scrambled siRNA or GLI1 siRNA for 48 h and changes in the expression of thyroid-specific proteins were evaluated. (**A**) Western blot analysis showing expression of thyroid-specific proteins (thyroperoxidase (TPO) and TSH receptor (TSHR)) and transcription factors (PAX-8 and TTF-1) in TPC-1 cells. (**B**) Quantitative analysis of western blots. GAPDH was used as an internal control. Mean ± SD values from at least three independent experiments are reported. * *p* < 0.05, ** *p* < 0.01 (Student’s *t*-test). (**C**) Changes of thyroid-specific proteins and transcription factors expression in SW1736 cells with GLI1 knockdown. (**D**) Quantitative analysis of thyroid-specific proteins and transcription factors expression in SW1736 cells. Mean ± SD values from at least three independent experiments are presented. * *p* < 0.05, *** *p* < 0.001 (Student’s *t*-test). Raw data is presented in Figures S11–S14.

We investigated the effect of GLI1 knockdown on the expression of thyroid-specific proteins and transcription factors. As shown in Figure 3A,B, treatment of GLI1 siRNA triggered increase of PAX-8 and TTF-1 expression compared to treatment with scrambled siRNA in TPC-1 cells (PAX-8: 2.77 ± 1.00; TTF-1: 1.47 ± 0.22). Moreover, the GLI1 siRNA treatment increased the expression of both TPO and TSHR in comparison with scrambled siRNA treatment (TPO: 1.47 ± 0.41; TSHR: 1.34 ± 0.17). SW1736 cells also showed robust effects of GLI1 knockdown for promoting expression of thyroid-specific proteins (TPO: 1.33 ± 0.21; TSHR: 3.82 ± 1.12) and transcription factors (PAX-8: 2.73 ± 0.75; TTF-1: 1.32 ± 0.26). These results suggest that knockdown of GLI1 activated the expression of thyroid-specific proteins and transcription factors.

### 3.4. Restoration of RAI Avidity and I-131-Mediated Cytotoxicity Effects Caused by GLI1 Knock Down

**Figure 4:**
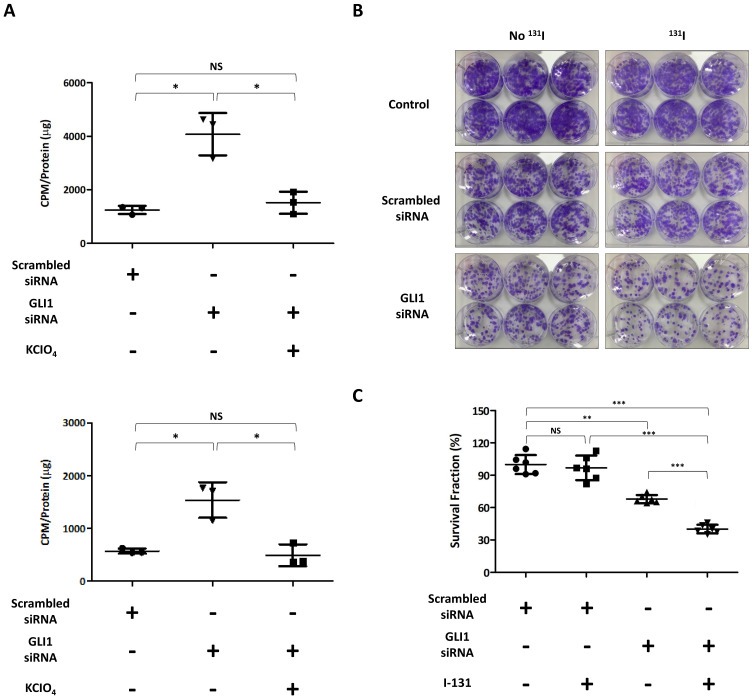
Verification of the extent of I-125 accumulation and I-131-mediated cytotoxic effect in GLI1-inhibited thyroid cancer cells. Both TPC-1 and SW1736 cells were treated with scrambled siRNA or GLI1 siRNA for 48 h. (**A**) For I-125 uptake assay, cells were treated with 37 kBq carrier-free I-125 and 100 μM sodium iodide at 37 °C for 30 min. Potassium perchlorate (KCIO_4_) was used as a competitive inhibitor of iodide transport. Upper–TPC-1 cells; Lower–SW1736 cells. The results are expressed as mean ± SD values of the experiment performed in triplicates. * *p* < 0.05, NS: Not Significant (by Student’s *t*-test). (**B**) After incubation of siRNA in TPC-1 cells, the cells were incubated with or without 50 µCi/ml I-131 supplemented with 30 μM NaI for 7 h at 37 °C. Images about I-131 clonogenic assay. (**C**) Quantitative analysis based on I-131 clonogenic assay. The results are expressed as mean ± SD values of the experiment performed in triplicates. *** *p* < 0.001, ** *p* < 0.01, NS: Not Significant (by Student’s *t*-test).

Next, we performed I-125 uptake assay to evaluate whether GLI1 knockdown-induced augmentation of functional thyroid-specific proteins results in recovery of RAI avidity. GLI1 knockdown significantly increased I-125 accumulation in TPC-1 cells (3.28-fold compared to treatment with scrambled siRNA) (Figure 4A, Upper). In the case of SW1736 cells, I-125 uptake was increased 2.69-fold by GLI1 knockdown than scrambled siRNA treatment. In addition, increased RAI uptake induced by GLI1 knockdown was completely blocked by potassium perchlorate, which competitively inhibits the active iodide transport in both cells. These results suggested that GLI1 knockdown in thyroid cancer cells could affect to restore RAI avidity through augmented expression of functional thyroid specific proteins and transcription factors.

After confirmation of recovery of RAI uptake via GLI1 knockdown, we further evaluated the effect of GLI1 knockdown on I-131-mediated cytotoxicity in TPC-1 cells, which showed high RAI avidity compared with SW1736 cells. As shown in Figure 4B, I-131 treatment of TPC-1 cells without GLI1 siRNA pre-treatment showed a trivial cytotoxic effect compared with the control group. Although the scrambled siRNA group also showed similar patterns as the control, it showed slight cytotoxic effect by siRNA transfection regardless of I-131 treatment. GLI1 siRNA transfection group showed marginal cytotoxic effect when compared with scrambled siRNA group without I-131 treatment (scrambled siRNA: 100% ± 8.87%; GLI1 siRNA: 67.97% ± 3.76%). Moreover, the I-131 treatment group with GLI1 siRNA pre-transfection showed highest cytotoxic effect with reduced formation of colonies compared with other groups (Scrambled siRNA: 100% ± 8.87%; Scrambled siRNA with I-131: 96.94% ± 11.38%; GLI1 siRNA: 67.97% ± 3.76%; GLI1 siRNA with I-131: 40.11% ± 3.99%; Figure 4C). These results indicate that knockdown of GLI1 also has a cytotoxic effect and that I-131-mediated cytotoxicity occurs via a blockade of GLI1 expression.

### 3.5. Identification of GLI1 Inhibitor Efficacy for NIS Expression

**Figure 5:**
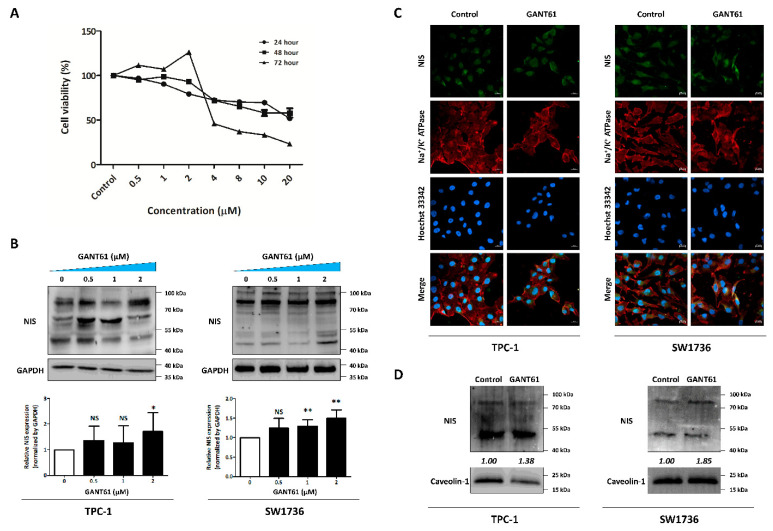
Efficacy of GANT61 in restoring endogenous NIS expression in thyroid cancer cells. Both TPC-1 and SW1736 cells were exposed to GANT61. (**A**) Results of cell viability assay showing time- and dose-dependent effects of GANT61 in TPC-1 cells. Mean ± SD values from three optical density (OD) is reported. (**B**) Endogenous NIS expression in whole cell lysate after treatment with GANT61. GAPDH was used as a loading control. The results are expressed as mean ± SD values of the experiment performed in quintuplicates. * *p* < 0.05, ** *p* < 0.01, NS: Not Significant (by Student’s *t*-test). (**C**) Immunofluorescence images for monitoring changes of expression and localization with endogenous NIS in thyroid cancer-derived cells treated with GANT61. (**D**) Change of endogenous NIS expression in plasma membrane proteins. Caveolin-1 were used as loading controls for plasma membrane proteins. Raw data is presented in Figures S15 and S16.

Based on the siRNA experiments, we assessed the efficacy of GANT61 (GLI1 inhibitor) in restoration of endogenous NIS expression. First, we confirmed the concentration- and time-dependent effect of GANT61 on cell viability in TPC-1 cells. The 24 h and 48 h incubation groups showed a gradual decrease in cell viability, reaching approximately 50% of viable cells at the highest concentration (24 h: 51.76 ± 1.79; 48 h: 57.97 ± 5.16; Figure 5A). In addition, cell viability sharply decreased from 4 μM concentration onwards in the 72 h group (23.14% ± 0.21% at the highest concentration). Therefore, we chose the suitable concentrations (0.5, 1 and 2 μM) associated with a reasonable range of cell viability (approximately 80–90%) with 24 h incubation to observe changes in NIS expression at no significant cytotoxic concentration. Next, we confirmed the restoration of endogenous NIS expression after treatment with GANT61. As shown in Figure 4B, GANT61 treatment increased the endogenous NIS expression in both cell lines. In the case of TPC-1 cells, NIS proteins at partial glycosylation (between 55 and 70 kDa) gradually increased dose-dependent (0.5 and 1 μM) after GANT61 treatment. However, its expression was maximized at 2 μM on full glycosylation (between 70 and 100 kDa). Quantitative analysis by measuring NIS protein bands between 70–100 kDa showed that both thyroid cancer cells significantly promoted the expression of endogenous NIS protein at 2 μM of GANT61. Next, we monitored the change of NIS expression and its localization by treating GANT61 using Immunofluorescence imaging for NIS protein. GANT61 treatment to thyroid cancer cells triggered an increase in endogenous NIS expression, as well as their localization on plasma membrane (Figure 5C). Western blot analysis also showed that endogenous NIS expression on the plasma membrane showed an increasing pattern in GANT61 group compared to the control. These results suggest that GANT61, a GLI1 inhibitor, promoted endogenous NIS expression, especially on the membrane.

## 4. Discussion

In the present study, we demonstrated that inhibition of GLI1 transcription factor to restore endogenous NIS expression can improve RAI avidity in thyroid cancer-derived cells, which increases the I-131-mediated cytotoxic effect.

Global cancer statistics have shown an increasing trend in the incidence of thyroid cancer and the associated mortality rates [18]. Generally, DTC has an excellent prognosis with multimodal treatment (10-year survival rate: approximately 90%) [19,20]. However, the 10-year survival rate of patients with RAI-refractory thyroid cancers with dedifferentiation status is <10%, mainly due to reduced RAI uptake and lack of response to RAI therapy [3,4]. Aberrant activation of MAPK and PI3K/AKT signaling pathways by point mutations or chromosomal rearrangements are fundamental drivers of tumorigenesis and RAI refractoriness in thyroid cancers [3]. These alterations are known to be associated with an aberrant silencing of thyroid-specific proteins related to iodide-metabolizing machinery, leading to a failure of RAI therapy [21,22]. Thus, many compounds have been used in an attempt to convert RAI-refractory thyroid cancers into RAI-sensitive thyroid cancers; however, no meaningful results have been obtained as yet [2]. Therefore, identification of novel targets for inducing redifferentiation with an improvement of RAI therapy is a key imperative.

We compared expression level of factors regulating the hedgehog signaling pathway between various thyroid cancer cells. Each cell line showed differential expression of factors suggesting that the type of thyroid cancer does not influence the activation of hedgehog signaling pathway. Of interest, all thyroid cancer cells consistently expressed the secretory SHH ligand, suggesting the existence of an autocrine SHH. In addition, expression of upstream to downstream hedgehog signaling pathway was variable in thyroid cancer cells. Therefore, we can assume that these thyroid cancer cells lines were influenced both canonical and non-canonical hedgehog signaling pathway. In a study by Xu et al., more than 65% of thyroid cancer specimens were positive for various factors related to SHH signaling pathways [23]. However, these factors showed no correlation with the thyroid cancer types or clinicopathologic parameters, suggesting extensive activation of SHH signaling pathway in thyroid cancers. In addition, a GLI1 transcription factor can be activated by various signaling pathways such as PI3K-Akt, MAPK, and Wnt/ß-catenin [24,25,26,27,28,29]. For example, Ji et al. demonstrated that oncogenic *KRAS V12* activates the GLI1 expression level and its transcriptional activity in human pancreatic ductal epithelial cells [26]. In addition, activation of mTOR/S6K1 signaling pathway was shown to promote the GLI1 transcriptional activity and oncogenic function [28]. Studies have also verified the crosstalk between MAPK and GLI1 in thyroid cancers; in addition, an MEK inhibitor inhibits GLI1 activation, which suggests the possibility of a non-canonical mechanism of hedgehog signaling pathway activation [15]. In our study, GLI1 expression showed that it is not correlated with factors regulating the hedgehog signaling pathway. This suggests that GLI1, as a transcription factor, may potentially be activated both canonically by hedgehog signaling pathways or non-canonically by other signaling pathways.

Several studies have suggested that the hedgehog signaling pathway is a potential therapeutic target in thyroid cancers [23,30,31]. In the study by Xu et al., knockdown of *SHH* and *GLI1* inhibited cell proliferation and cell cycle in aggressive thyroid cancers [23]. In addition, blockade of hedgehog signaling pathway by SMO antagonist also inhibited cell viability, inducing apoptosis in medullary thyroid cancers [31]. In the present study, knockdown of *GLI1* alone decreased the colony-forming ability of thyroid cancer cells, which is consistent with the previous studies. Moreover, a I-131-mediated cytotoxic effect was increased by pre-treatment with GLI1 siRNA and declined the colony-forming ability of thyroid cancer cells.

Even though several studies have demonstrated the role of the hedgehog-signaling pathway in inhibiting cell proliferation and apoptosis [23,30], restoration of RAI avidity with redifferentiation by modulating the hedgehog signaling pathway has not yet been investigated in thyroid cancers. Our ultimate goal was to induce conversion from dedifferentiation status to redifferentiation status with an increase in both thyroid-specific proteins and transcription factors by inhibiting the GLI1 transcription factor, thereby restoring RAI uptake in dedifferentiated thyroid cancers. Therefore, we evaluated the potential use of the GLI1 transcription factor inhibition for promoting redifferentiation in thyroid cancer. Our results showed that inhibition of the GLI1 transcription factor led to an increased expression of both thyroid-specific proteins and transcription factors, and an improvement of the I-131-mediated cytotoxic effect.

In our study, knockdown of GLI1 increased endogenous NIS expression, especially the plasma membrane fraction in thyroid cancer cells. Indeed, a lack of endogenous NIS functionality is not restricted to decreased NIS expression, but it can result from diminished membrane targeting or insufficient retention of NIS on the membrane [20]. Approximately 70% of thyroid cancers were shown to express or even overexpress NIS in immunohistochemical profiles [32,33]. However, expression of NIS was predominantly intracellular not on membrane. In a comparative study, tumors harboring *BRAF^V600E^* mutation showed a significantly low NIS expression and impaired targeting to membrane in a series of 60 human PTC samples [34]. Besides, several factors, including blockade of phosphatidylinositol glycan anchor biosynthesis class U (PIGU) by activating an MAPK signaling pathway, increased leukemia-associated RhoA guanine exchange factor (LARG) owing to PTEN deficiency, induction of NIS proteolysis by valosin-containing protein as a component of endoplasmic reticulum-associated degradation, and overexpression of pituitary tumor-transforming gene 1 (PTTG1)-binding factor (PBF), have been shown to trigger impairment of NIS membrane targeting [35,36,37,38,39,40,41]. On the other hand, recognition of ADP-ribosylation factor 4 (ARF4) by VAPK motif in the NIS C-terminus, regulation of SRC/RAC1/PAK1/PIP5K/EZRIN pathway, activation of RAC1 via MAPK inhibition, application of transient receptor potential vanilloid type 1 (TRPV1) agonist and others were shown to promote NIS functionality and trafficking towards the cell membrane [41,42,43,44,45]. Recently, an article suggested the role of protospacer adjacent motif (PDZ)—PDZ interaction which means PDZ-domain containing protein SCRIB binds to the carboxy-terminus of NIS to localize at proper basolateral plasma membrane [46]. These findings indicate that membrane targeting of NIS is also important, as much as an increase in NIS expression to revert RAI-refractory thyroid cancer into RAI-sensitive thyroid cancer. Therefore, NIS must be expressed, targeted, and retained on the plasma membrane of thyroid cells for successful RAI therapy.

GLI antagonists, or GANTs, were discovered at the National Cancer Institute and identified in a cell-based screen with HEK293 cells transiently expressing GLI1 and a GLI-dependent luciferase reporter assay [47]. Both GANT58 and GANT61 were shown to inhibit GLI-mediated gene activation; however, GANT61 is more specific toward GLI proteins and more effectively reduces the GLI1 and GLI2 DNA-binding ability. In addition, GANT61 has shown the inhibitory effect on the growth of several cancers, including ovarian cancer and neuroblastoma [14,48,49]. In thyroid cancer, treatment with GANT61 was shown to induce autophagy and to suppress tumor growth with apoptosis [50,51,52]. In addition, it was shown to affect the crosstalk with other signaling pathways, such as PI3K/AKT and TGF-β-activated kinase (TAK1) [51,52]. However, the effects of GANT61 for inducing redifferentiation in thyroid cancer are not known. We first evaluated and confirmed the efficacy of GANT61 in increasing NIS expression in thyroid cancer cells. Interestingly, arsenic trioxide, which is an FDA-approved inhibitor of GLI1 and GLI2 transcription factors, is used as a single agent against acute promyelocytic leukemia [53,54]. Moreover, arsenic trioxide has been used as a radio-sensitizing agent against breast cancer, fibrosarcoma cells, and glioblastoma [55,56,57,58,59]. Fröhlich et al. demonstrated the effectiveness of arsenic trioxide for promoting iodide uptake in thyroid cancer cells. However, there was no results about the change of thyroid specific proteins expression, I-131-mediated cytotoxicity effect and accurate molecular mechanisms following arsenic trioxide treatment was not elucidated [60,61]. Therefore, additional studies of GANT61 and arsenic trioxide including association with GLI1 may facilitate a better understanding of the roles of hedgehog signaling pathway with respect to redifferentiation of RAI-refractory thyroid cancers.

Even though many candidate drugs have shown meaningful results with respect to overcoming dedifferentiation in RAI-refractory thyroid cancers, no significant clinical benefits with pharmacologic interventions have been reported. Most studies have focused on specific signaling pathways, but not on comprehensive multiple signaling pathways that are involved in the causation of RAI-refractoriness. Therefore, strategies involving combined treatment targeting comprehensive signaling pathways may be applied to obtain synergistic effects. For instance, Montero-Conde et al. demonstrated that the BRAF inhibitor promotes the expression and activation of human epidermal growth factor receptor (HER)2/HER3 heterodimers depending on autocrine neuregulin-1 (NRG-1) binding to HER3 in thyroid cancers harboring *BRAF* mutation [62]. It triggered HER3 receptor phosphorylation followed by reactivation of PI3K/AKT and MAPK signaling pathways, and induction of drug resistance. Cheng et al. reported that the application of HER inhibitor prevented the rebound of MAPK signaling pathway and induced re-sensitization to BRAF/MEK inhibitor with redifferentiation and increased RAI uptake [63]. Besides, several trials (such as trials of BRAF inhibitor plus MEK inhibitor, HDAC inhibitor plus BRAF/MEK inhibitor) have also demonstrated synergistic effects in inducing redifferentiation [2,21,64,65]. Therefore, GLI1 inhibitor may potentially be added to other redifferentiation strategies to overcome therapeutic resistance or to obtain synergistic effects in RAI-refractory thyroid cancers.

## 5. Conclusions

Our findings demonstrated that inhibition of GLI1 could restore thyroid-specific proteins and transcription factors related to iodide-metabolizing machinery and promote redifferentiation in thyroid cancer cells. Furthermore, GLI1 inhibition improved RAI uptake and promoted I-131-mediated cytotoxic effect. Therefore, GLI1, as a novel target for inducing redifferentiation, is a potential alternative strategy to convert RAI-refractory thyroid cancers to RAI-sensitive thyroid cancers.

## Data Availability

Data will be made available upon reasonable request to corresponding author.

## Supplementary Materials

The following supporting information can be downloaded at: https://www.mdpi.com/article/10.3390/cancers14071782/s1, Figure S1: Evaluation of GLI1 knockdown dose-dependent manner in TPC-1 cells, and immunofluorescence images by treating Scrambled siRNA, GLI1 siRNA and NIS siRNA.; Figure S2: Raw data of Figure 1A; Figure S3: Raw data of Figure 1B; Figure S4: Raw data of Figure 1C; Figure S5: Raw data of Figure 2A; Figure S6: Raw data of Figure 2B; Figure S7: Raw data of Figure 2C; Figure S8: Raw data of Figure 2D; Figure S9: Raw data of Figure 2G; Figure S10: Raw data of Figure 2H; Figure S11: Raw data of Figure 3A; Figure S12: Raw data of Figure 3B; Figure S13: Raw data of Figure 3C; Figure S14: Raw data of Figure 3D; Figure S15: Raw data of Figure 5B; Figure S16: Raw data of Figure 5D; Scheme S1: List of used primary antibodies for western blot analysis.
