# Supplementary material for: Targeting GLI1 Transcription Factor for Restoring Iodine Avidity with Redifferentiation in Radioactive-Iodine Refractory Thyroid Cancers
Source: Cancers (Basel). 2022 Mar 31;14(7):1782. doi: 10.3390/cancers14071782 (PMC8997411; doi:10.3390/cancers14071782)
Supplement: Supplementary file 1 [file cancers-14-01782-s001.zip › cancers-1638255-supplementary.pdf]

cancers

Supplementary Material: Targeting GLI1 Transcription Factor for Restoring Iodine Avidity with Redifferentiation in Radioactive-Iodine Refractory Thyroid Cancers

Ji Min Oh, Ramya Lakshmi Rajendran, Prakash Gangadaran, Chae Moon Hong, Ju Hye Jeong, Jaetae Lee and Byeong-Cheol Ahn *

Supplemental Materials

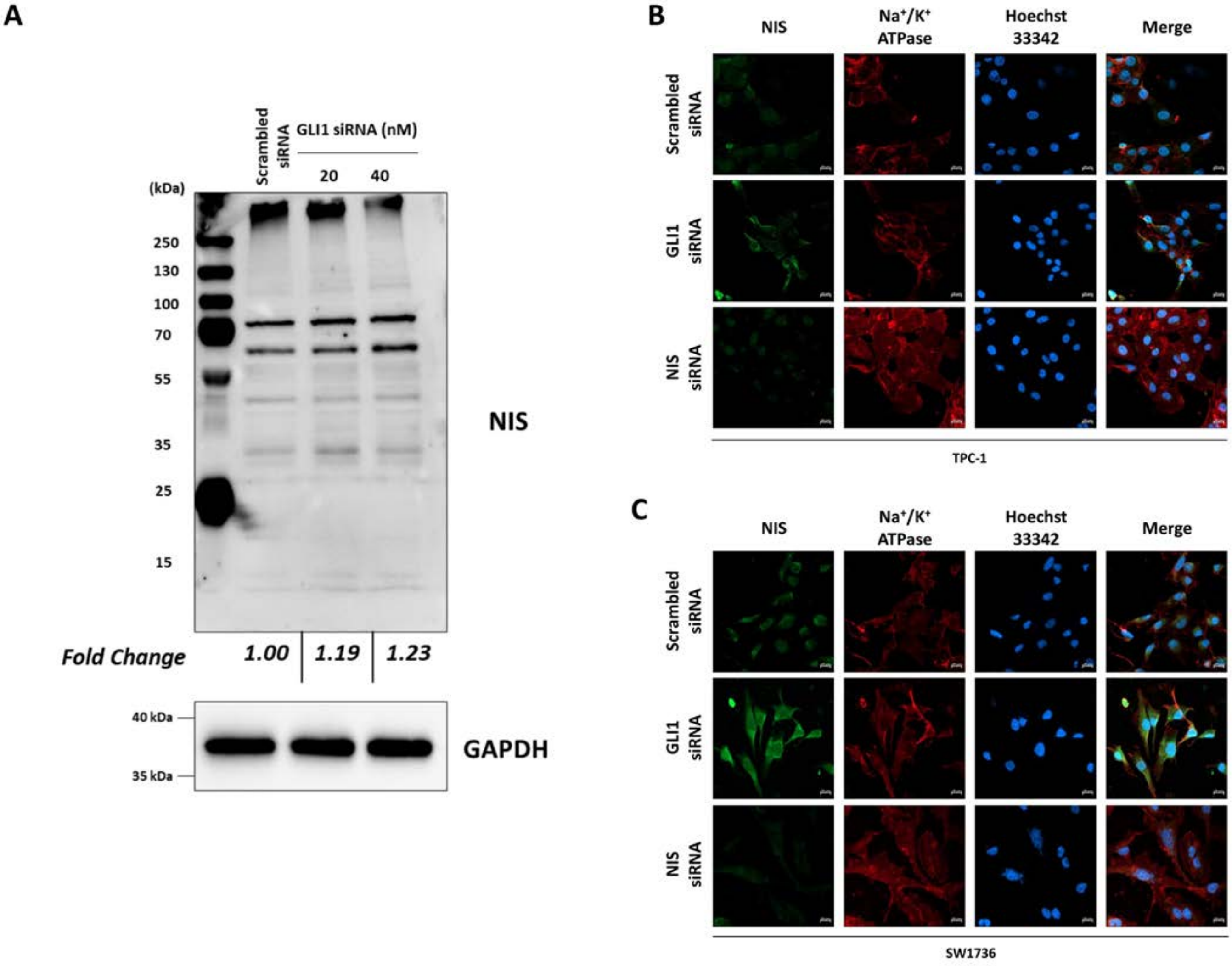

Figure S1. Thyroid cancer-derived cells were treated with scrambled siRNA, GLI1 siRNA or NIS siRNA for 48 hours. (A) Western blot analysis showing dose-dependent endogenous NIS expression after treatment with GLI1 siRNA in TPC-1 cells. Immunofluorescence images for endogenous NIS expression after scrambled, GLI1 or NIS siRNA treatment in TPC-1 cells (B) and SW1736 cells (C). Scale bar: 20μm

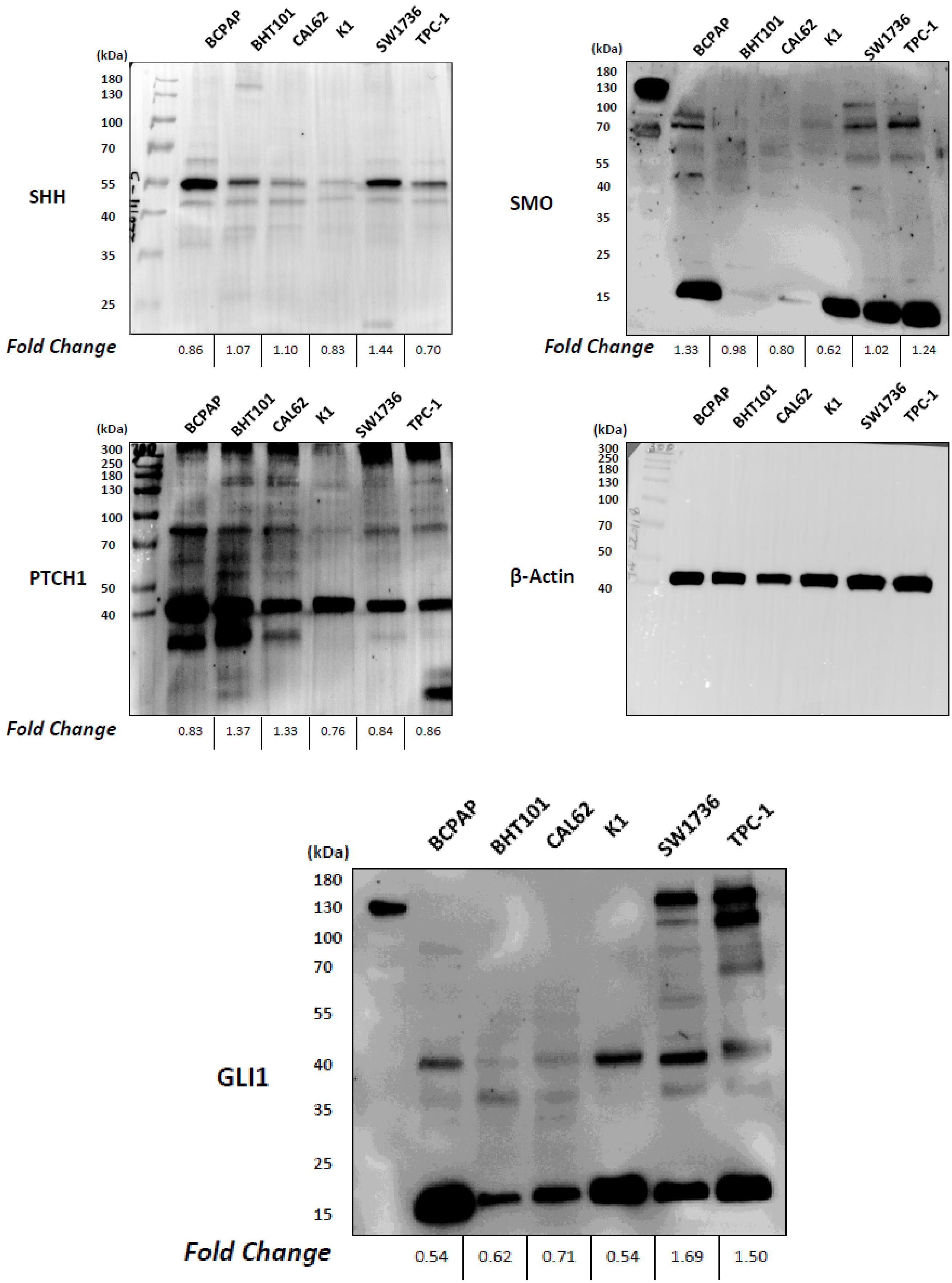

Figure S2. Raw data of Figure 1A.

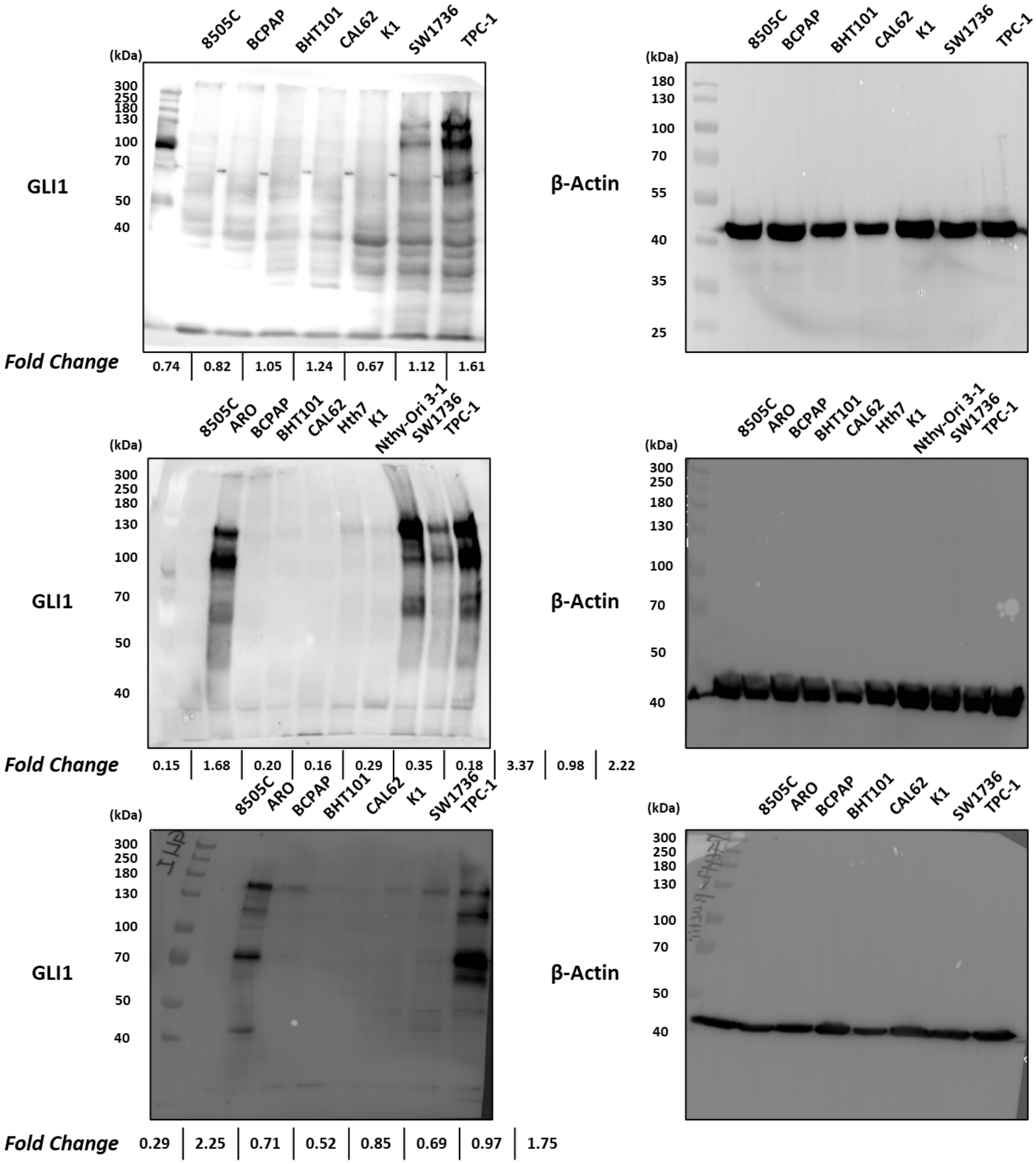
8505C BCPAP BHT101 CAL62 K1 SW1736 TPC-1
(kDa)
300 250 180 130 100 70 50 40
GLI1
Fold Change
0.74 0.82 1.05 1.24 0.67 1.12 1.61
8505C BCPAP BHT101 CAL62 K1 SW1736 TPC-1
(kDa)
180 130 100 70 55 40 35 25
β-Actin
8505C ARO BCPAP BHT101 CAL62 Hth7 K1 Nthy-Ori 3-1 SW1736 TPC-1
(kDa)
300 250 180 130 100 70 50 40
GLI1
Fold Change
0.15 1.68 0.20 0.16 0.29 0.35 0.18 3.37 0.98 2.22
8505C ARO BCPAP BHT101 CAL62 Hth7 K1 Nthy-Ori 3-1 SW1736 TPC-1
(kDa)
300 250 180 130 100 70 50 40
β-Actin
8505C ARO BCPAP BHT101 CAL62 K1 SW1736 TPC-1
(kDa)
300 250 180 130 100 70 50 40
GLI1
Fold Change
0.29 2.25 0.71 0.52 0.85 0.69 0.97 1.75
8505C ARO BCPAP BHT101 CAL62 K1 SW1736 TPC-1
(kDa)
300 250 180 130 100 70 50 40
β-Actin

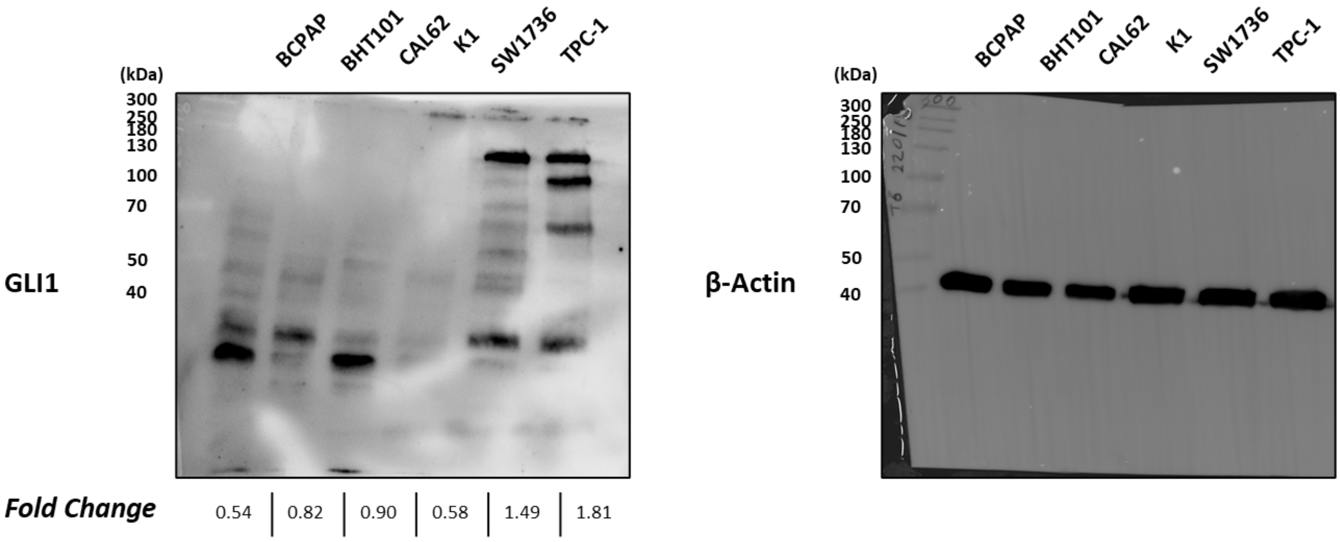

Figure S3. Raw data of Figure 1B.

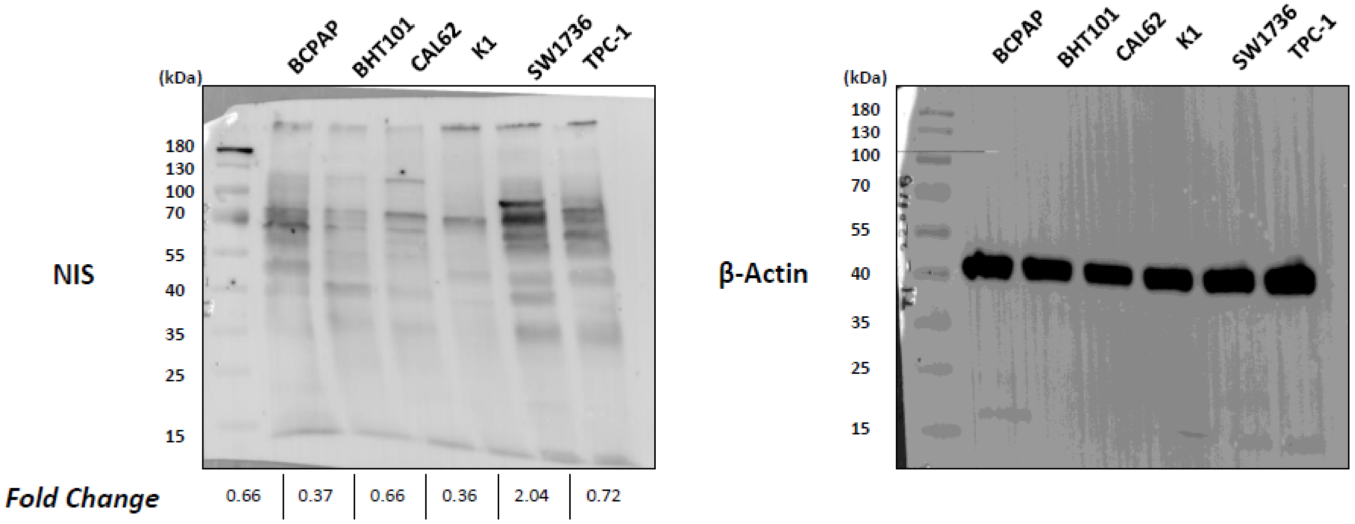

Figure S4. Raw data of Figure 1C.

TPC-1

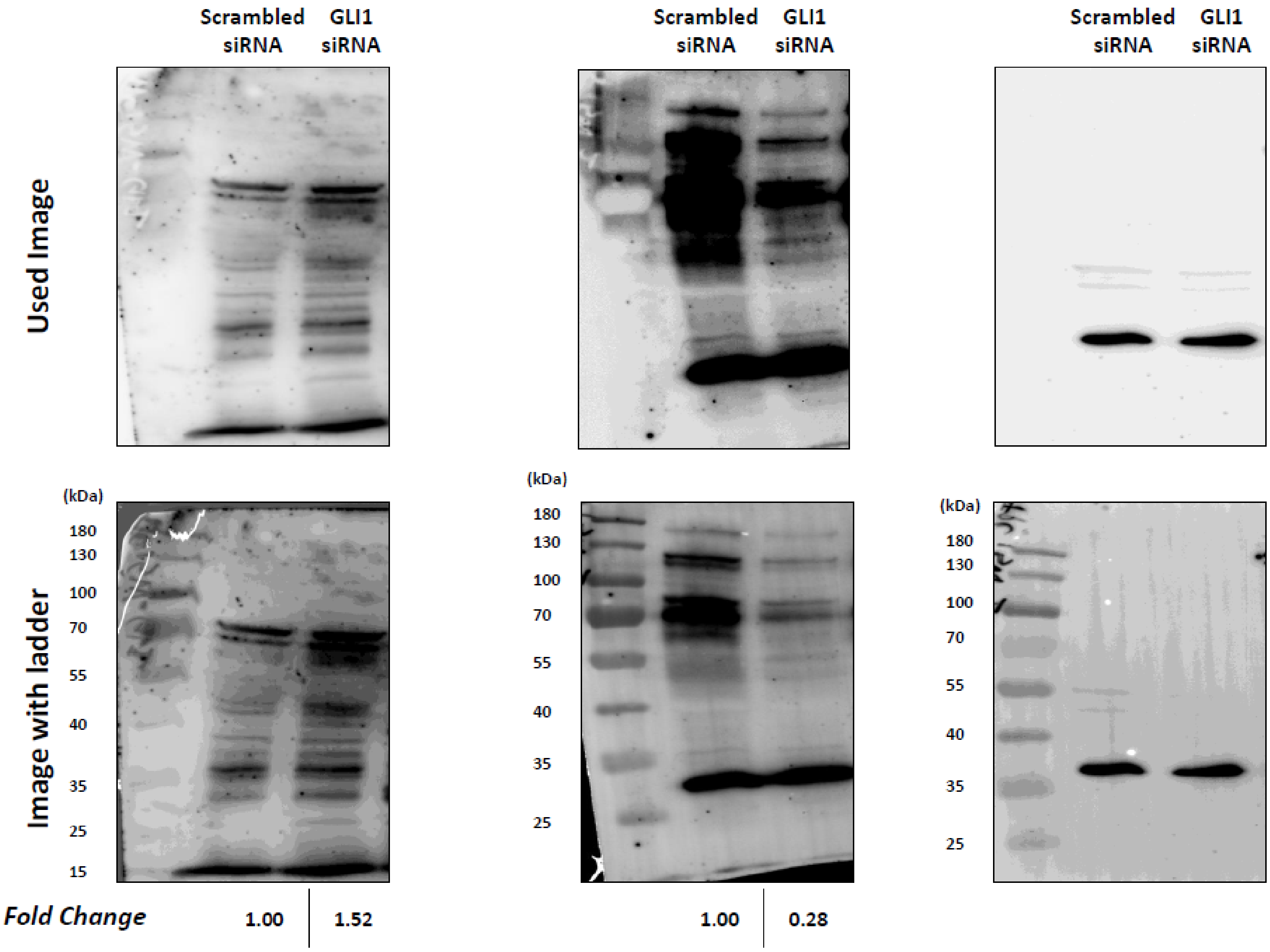

Scrambled siRNA
GLI1 siRNA
Scrambled siRNA
GLI1 siRNA
Scrambled siRNA
GLI1 siRNA
Used Image
Image with ladder
(kDa)
180
130
100
70
55
40
35
25
15
(kDa)
180
130
100
70
55
40
35
25
(kDa)
180
130
100
70
55
40
35
25
Fold Change
1.00
1.52
1.00
0.28

TPC-1

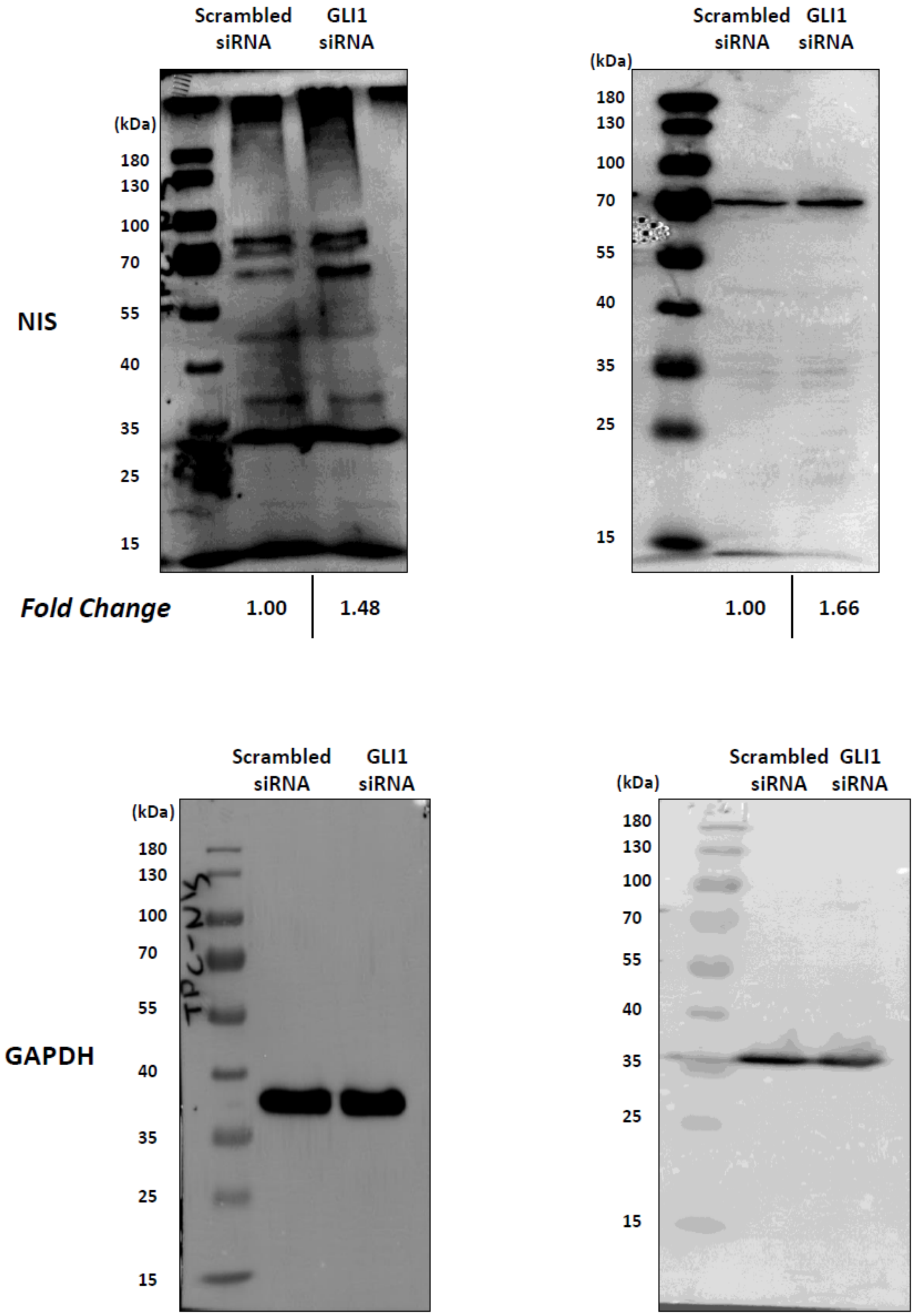
Scrambled siRNA
GLI1 siRNA
(kDa)
180
130
100
70
55
40
35
25
15
NIS
Fold Change
1.00
1.48
1.00
1.66
GAPDH

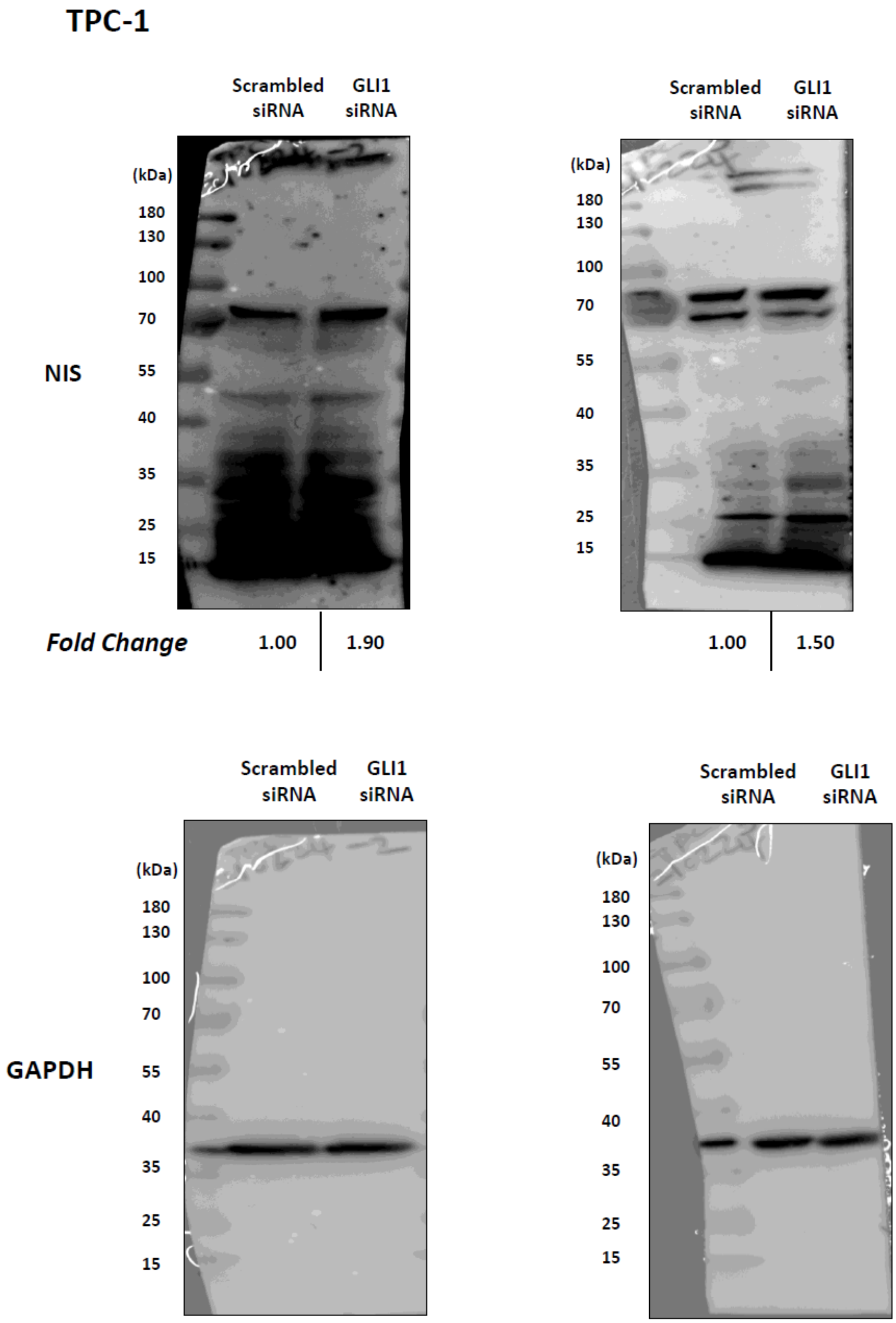

Figure S5. Raw data of Figure 2A.

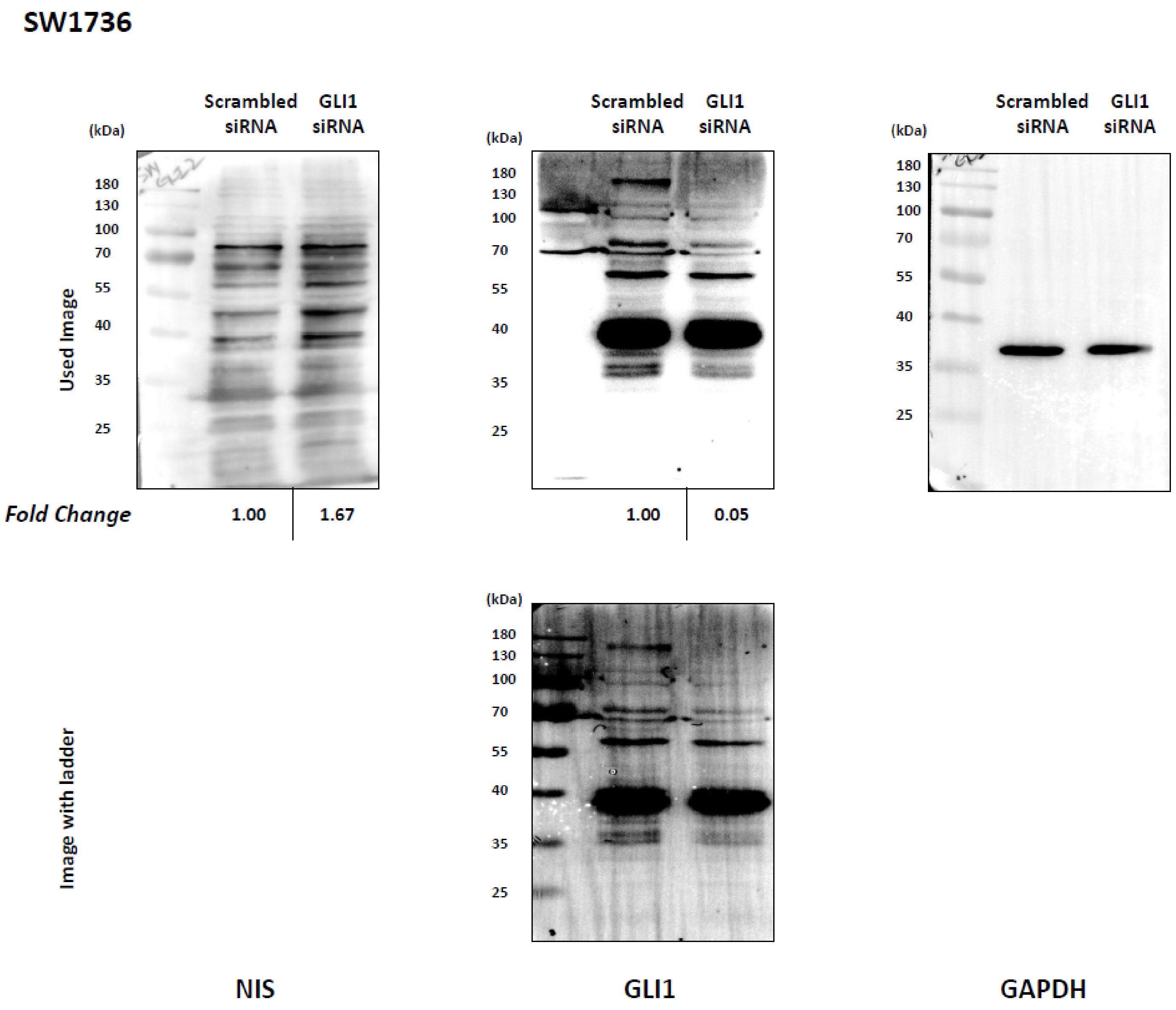

SW1736
Scrambled siRNA
GLI1 siRNA
(kDa)
180
130
100
70
55
40
35
25
Used Image
Image with ladder
Fold Change
1.00
1.67
1.00
0.05
NIS
GLI1
GAPDH

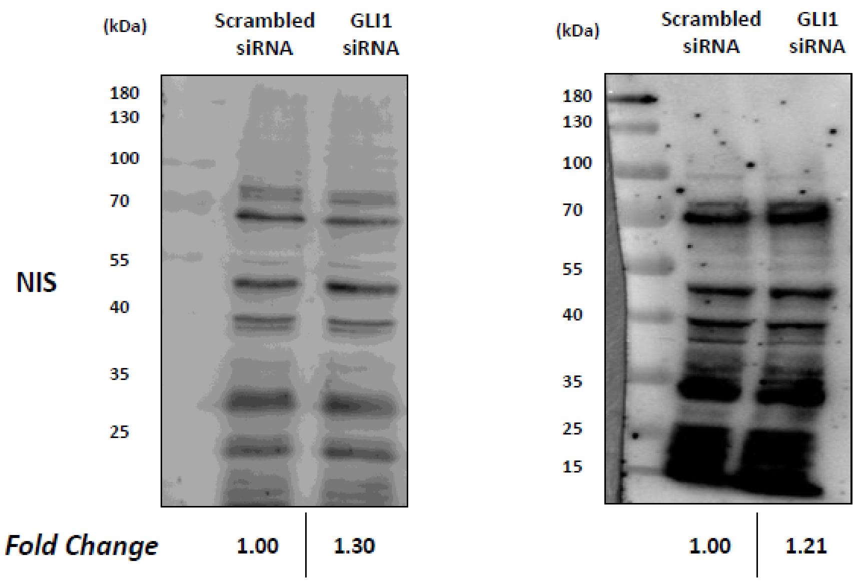

(kDa)
Scrambled siRNA
GLI1 siRNA
180
130
100
70
55
40
35
25
15
NIS
Fold Change
1.00
1.30
1.00
1.21

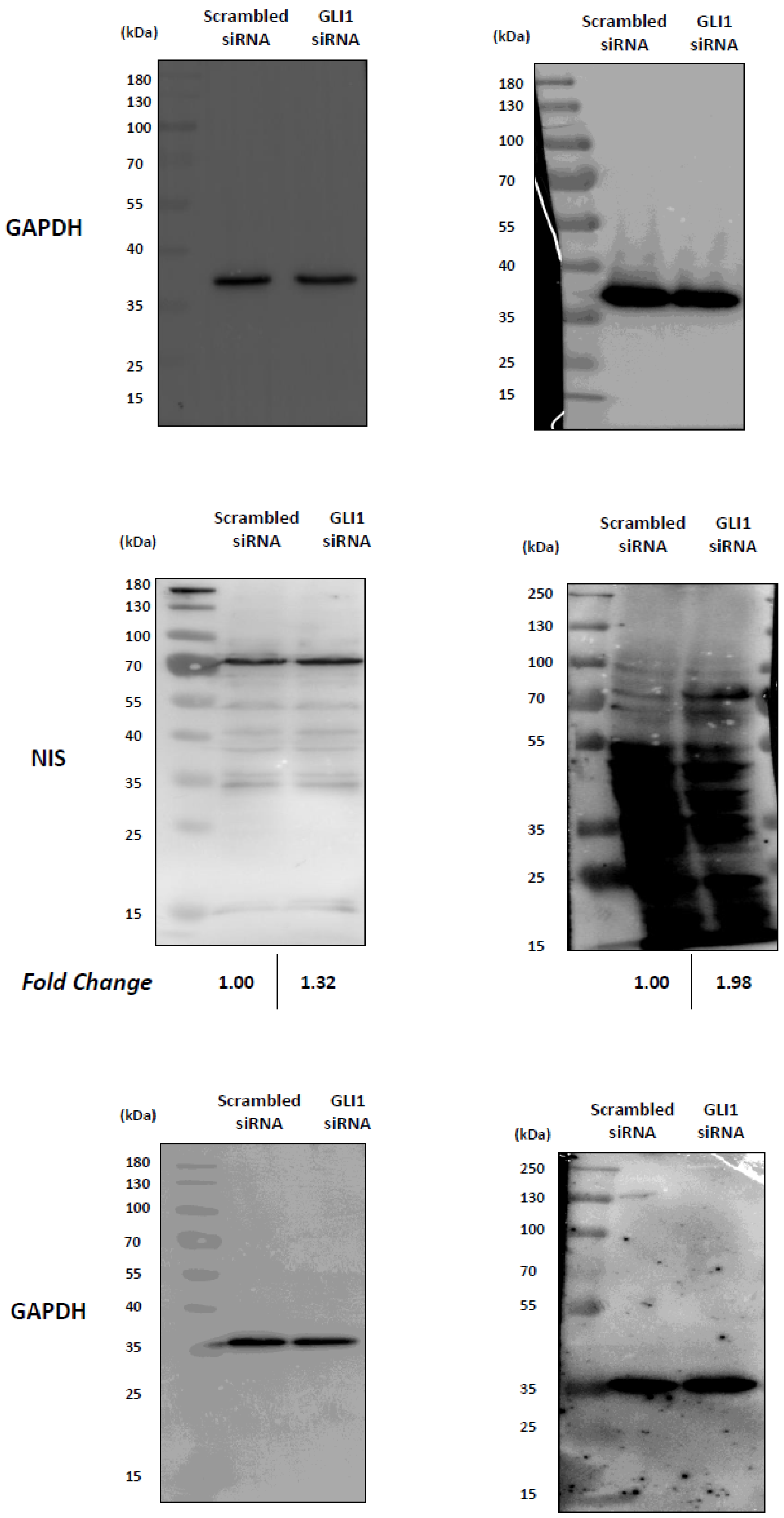

Figure S6. Raw data of Figure 2B.

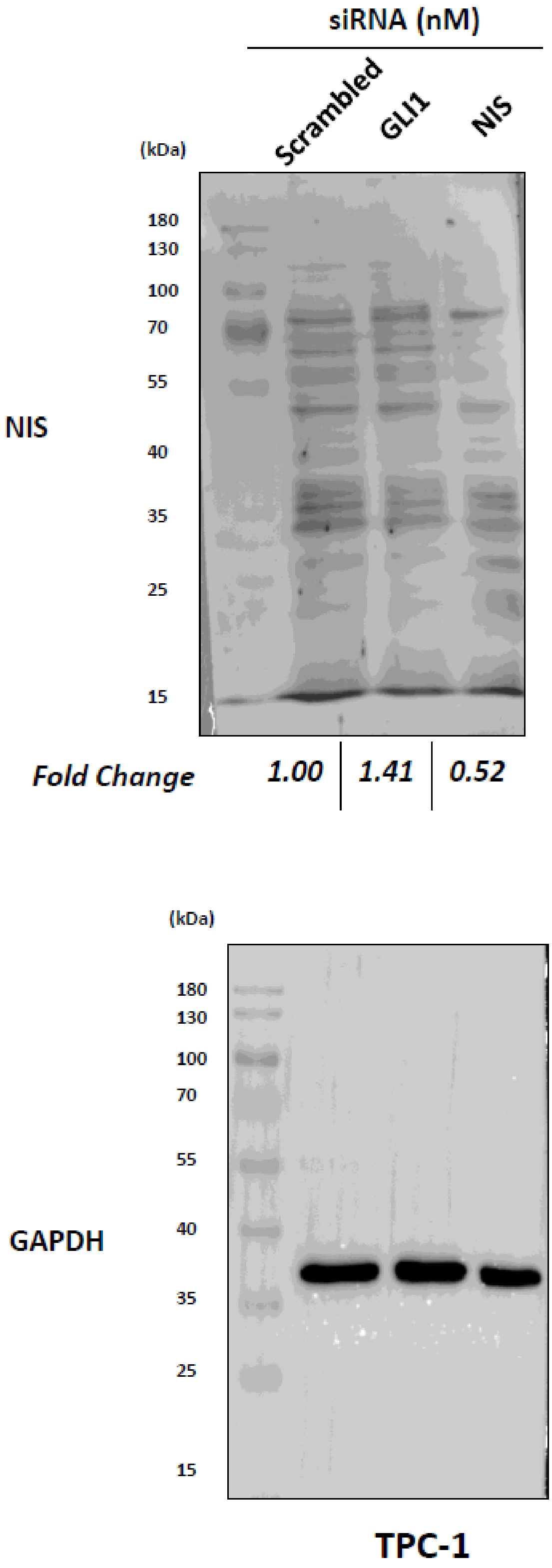

Figure S7. Raw data of Figure 2C.

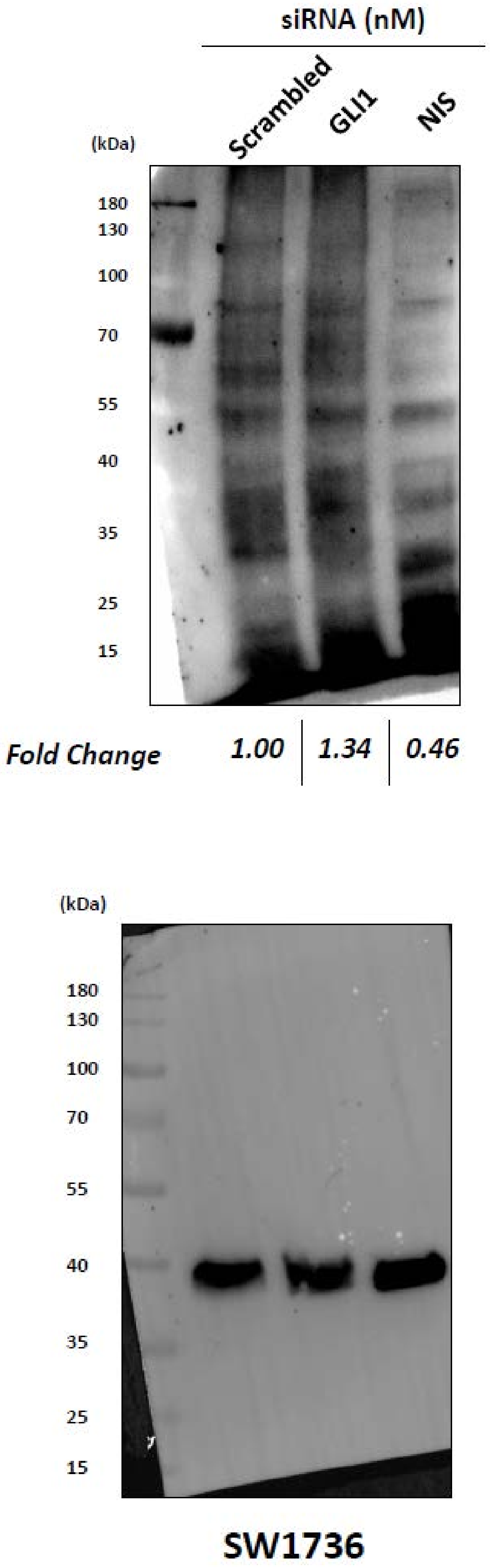

Figure S8. Raw data of Figure 2D.

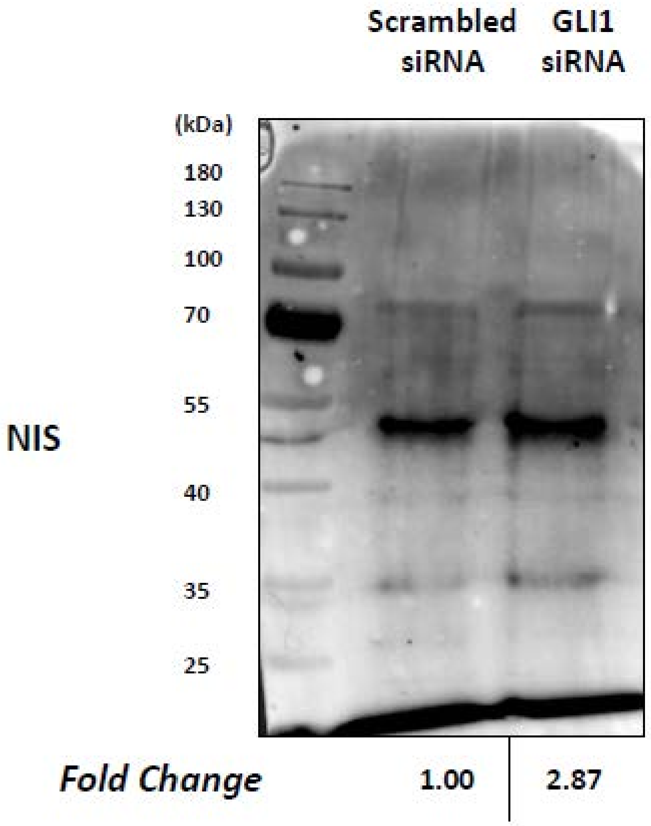

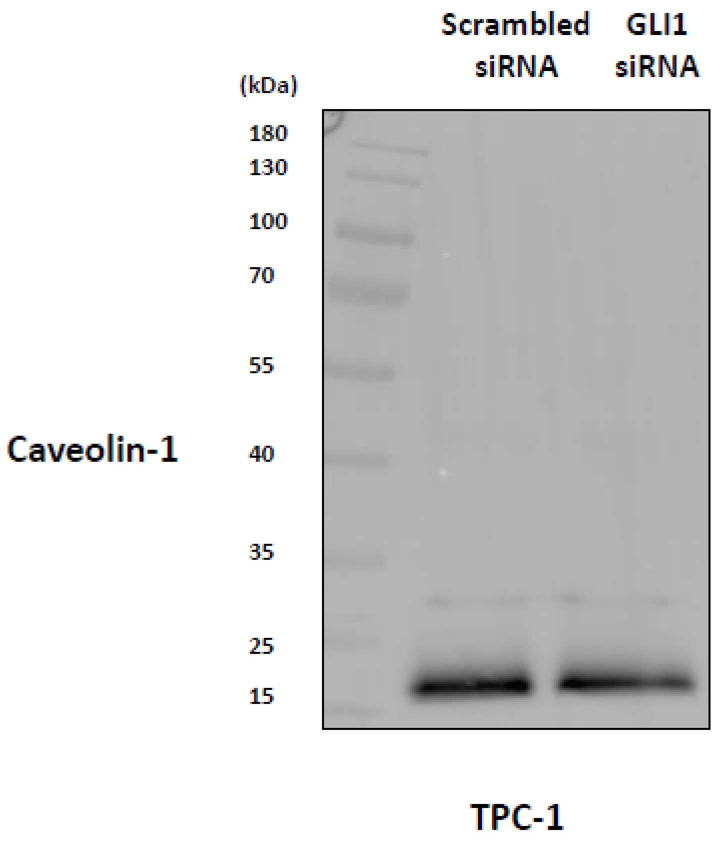

Figure S9. Raw data of Figure 2G.

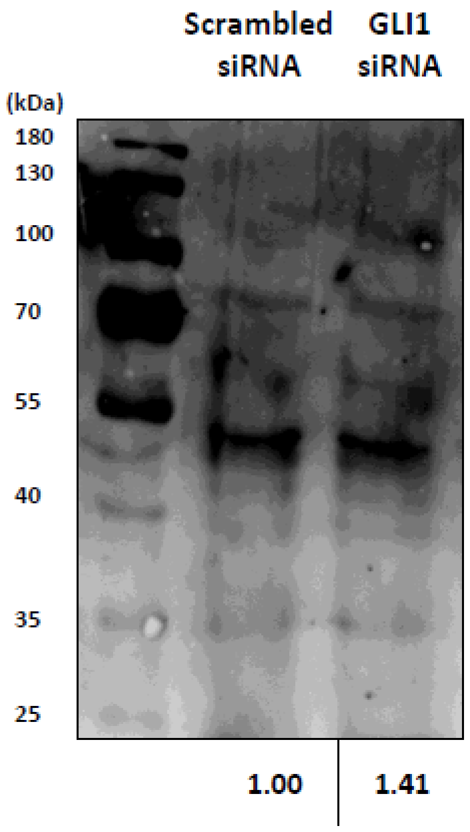

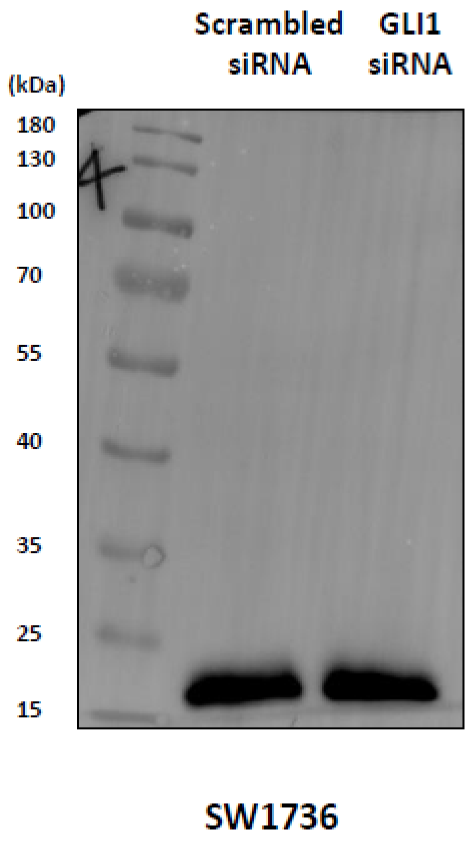

Figure S10. Raw data of Figure 2H.

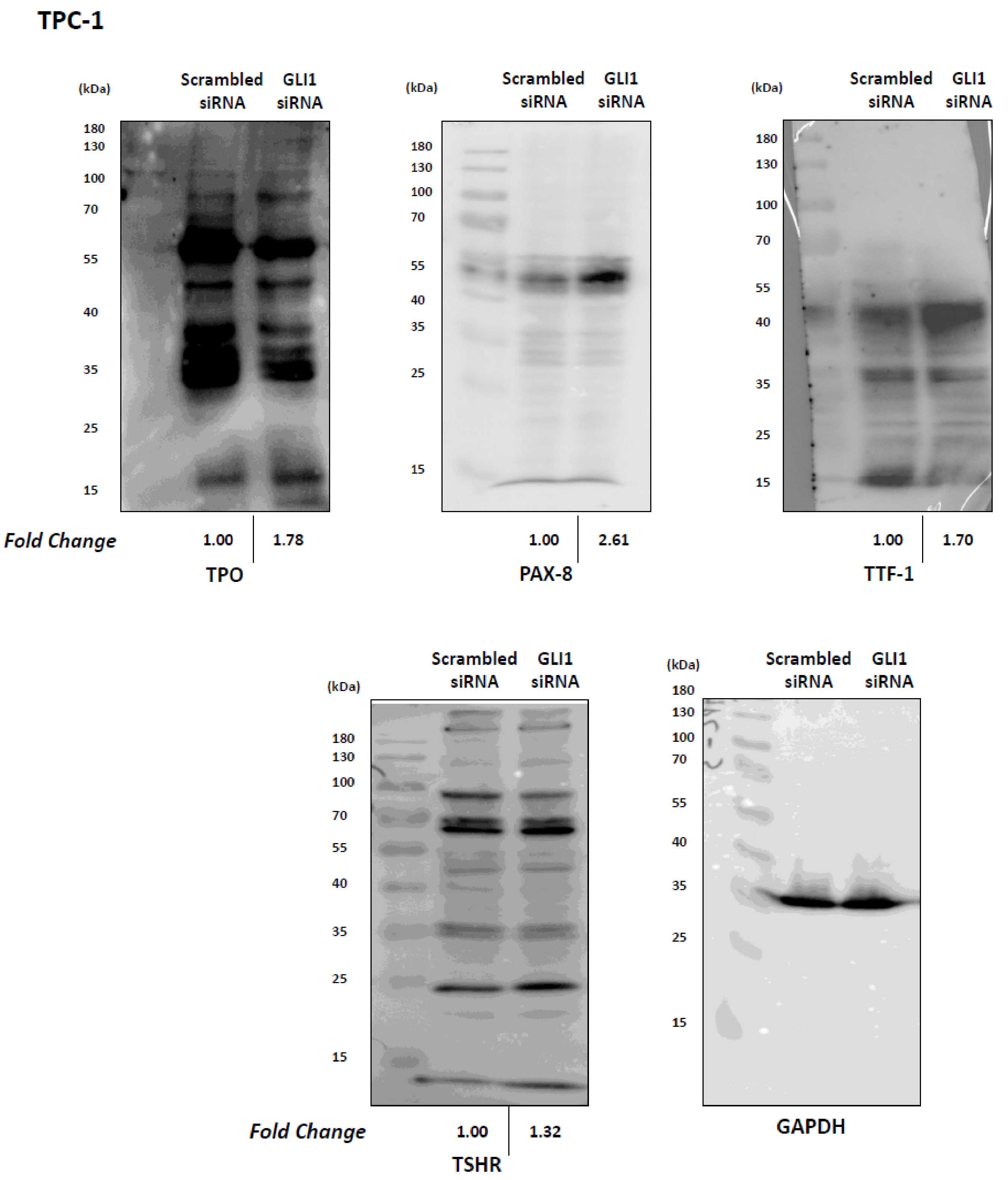

Figure S11. Raw data of Figure 3A.

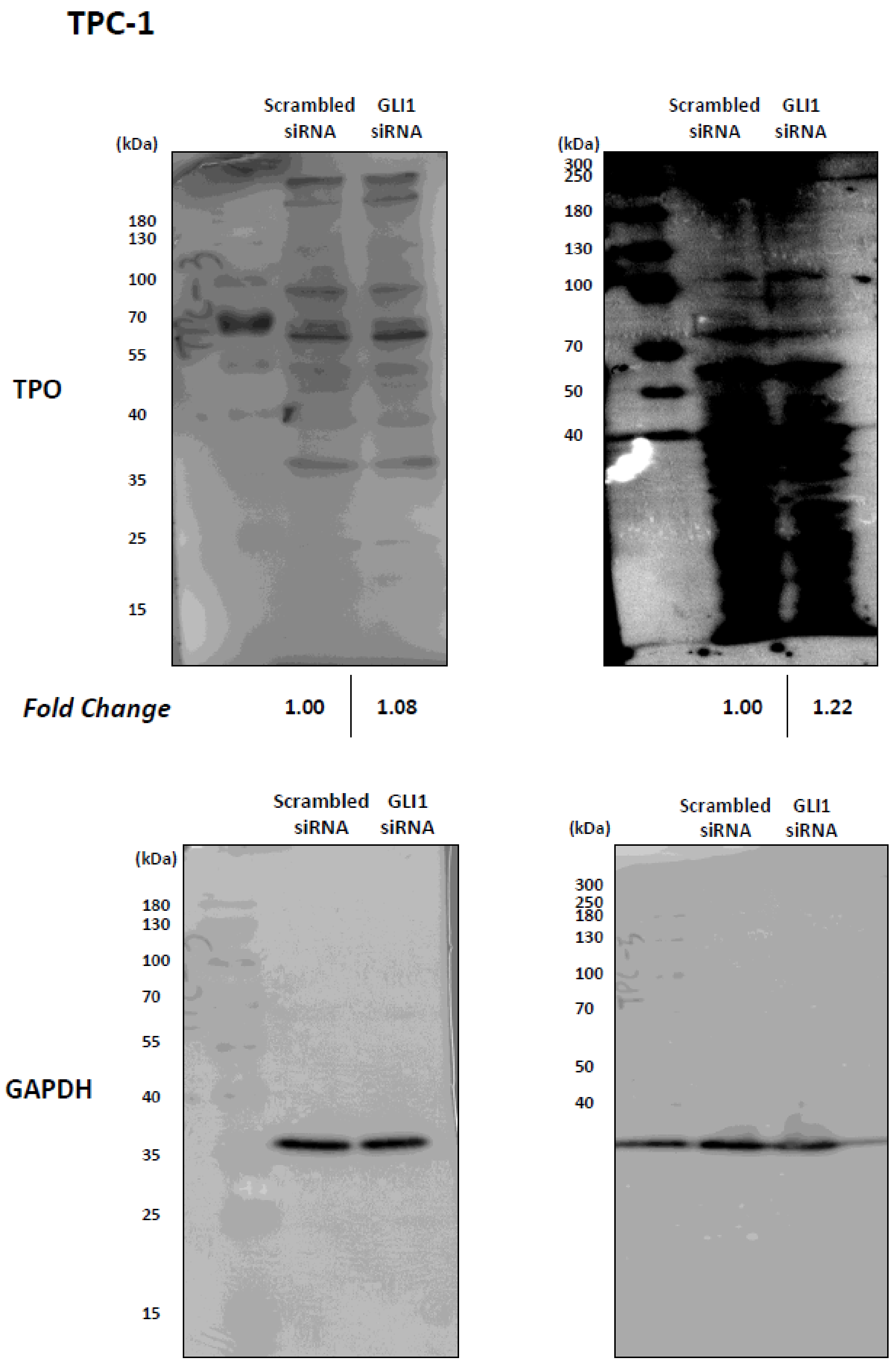
TPC-1
Scrambled siRNA
GLI1 siRNA
(kDa)
180
130
100
70
55
40
35
25
15
TPO
Scrambled siRNA
GLI1 siRNA
(kDa)
300
250
180
130
100
70
50
40
Fold Change
1.00
1.08
1.00
1.22
Scrambled siRNA
GLI1 siRNA
(kDa)
180
130
100
70
55
40
35
25
15
GAPDH
Scrambled siRNA
GLI1 siRNA
(kDa)
300
250
180
130
100
70
50
40

TPC-1

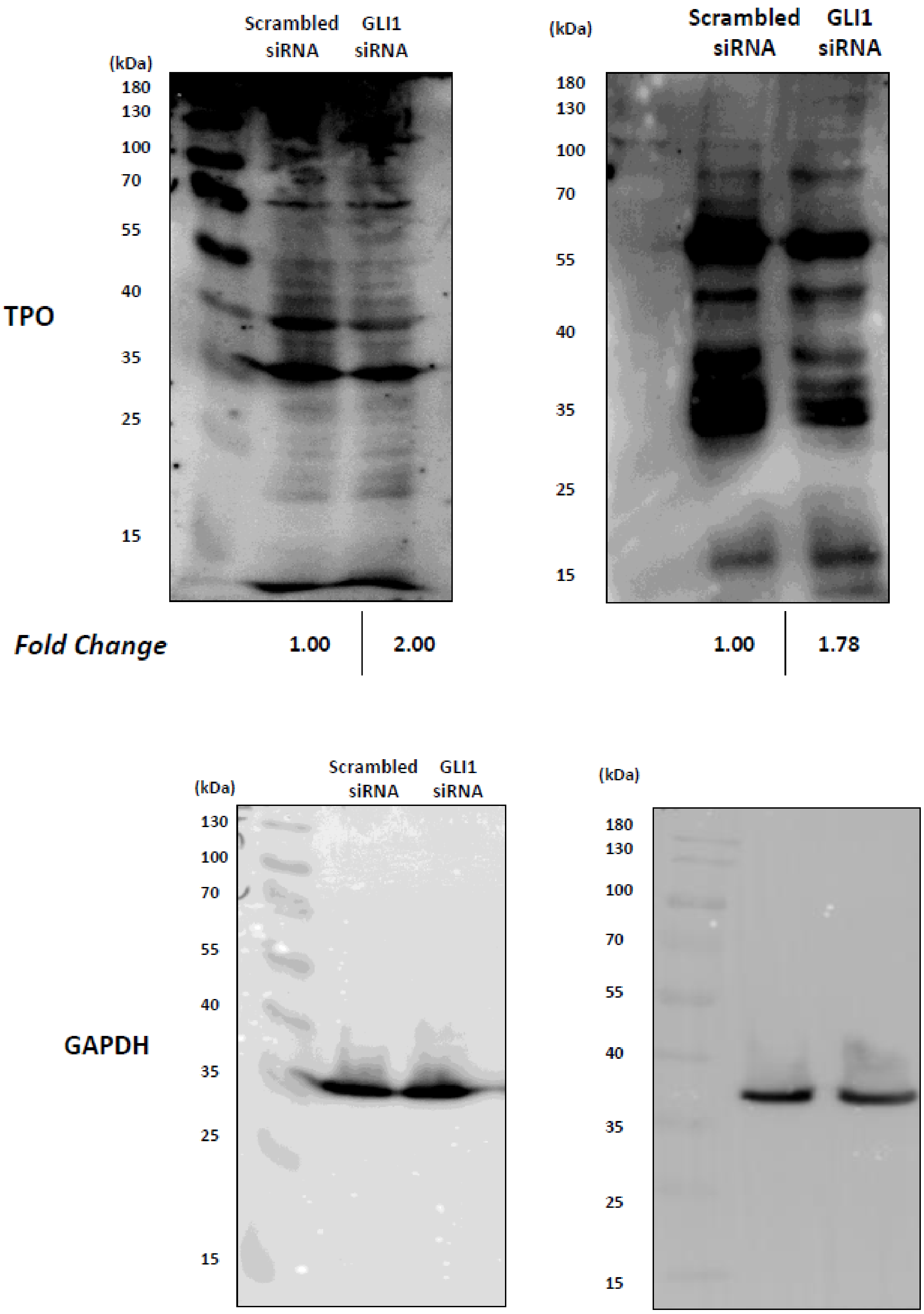
Scrambled siRNA
GLI1 siRNA
(kDa)
180
130
100
70
55
40
35
25
15
TPO
Fold Change
1.00
2.00
1.00
1.78
GAPDH

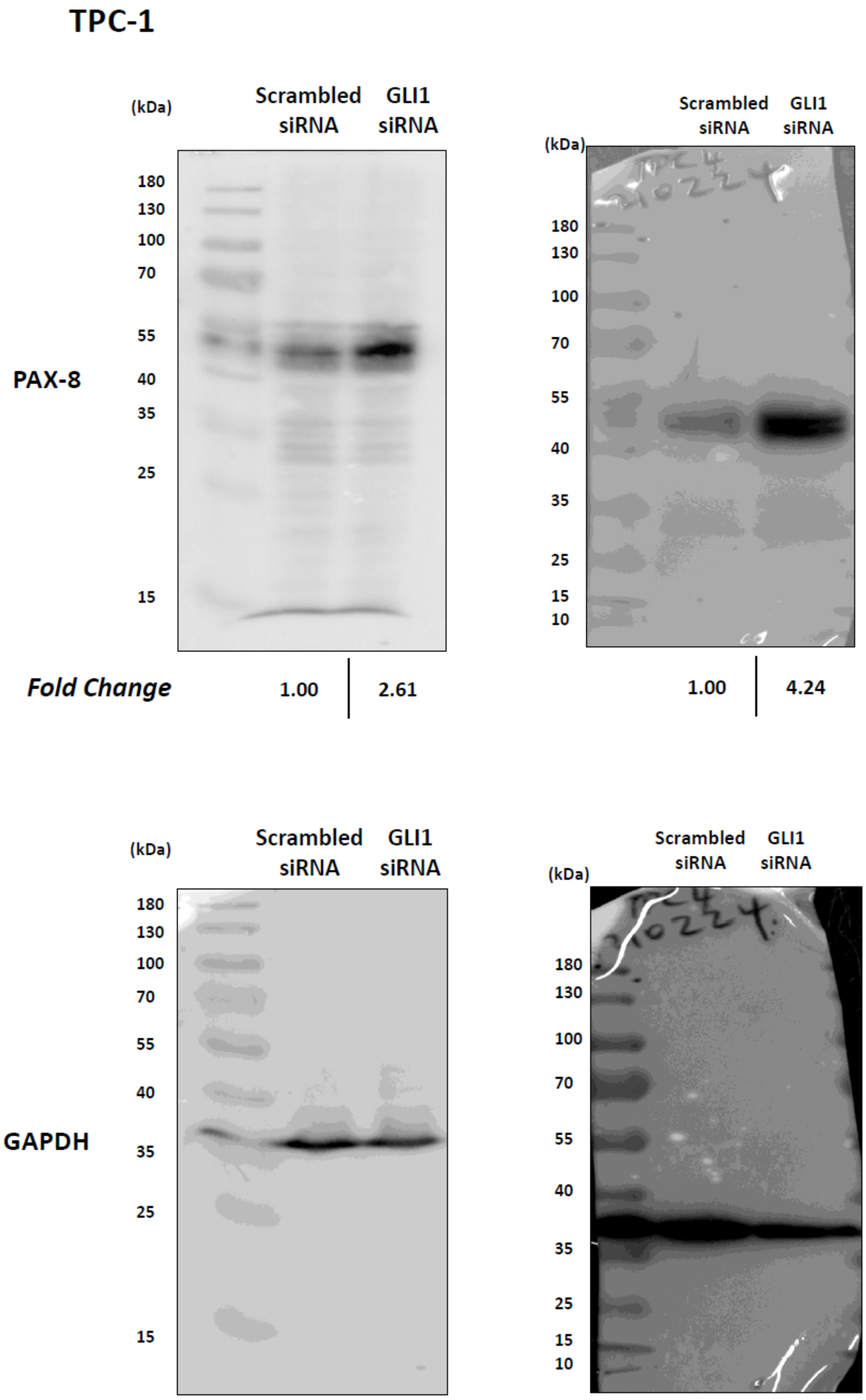

TPC-1
(kDa)
Scrambled siRNA
GLI1 siRNA
180
130
100
70
55
40
35
25
15
10
PAX-8
Fold Change
1.00
2.61
1.00
4.24
GAPDH

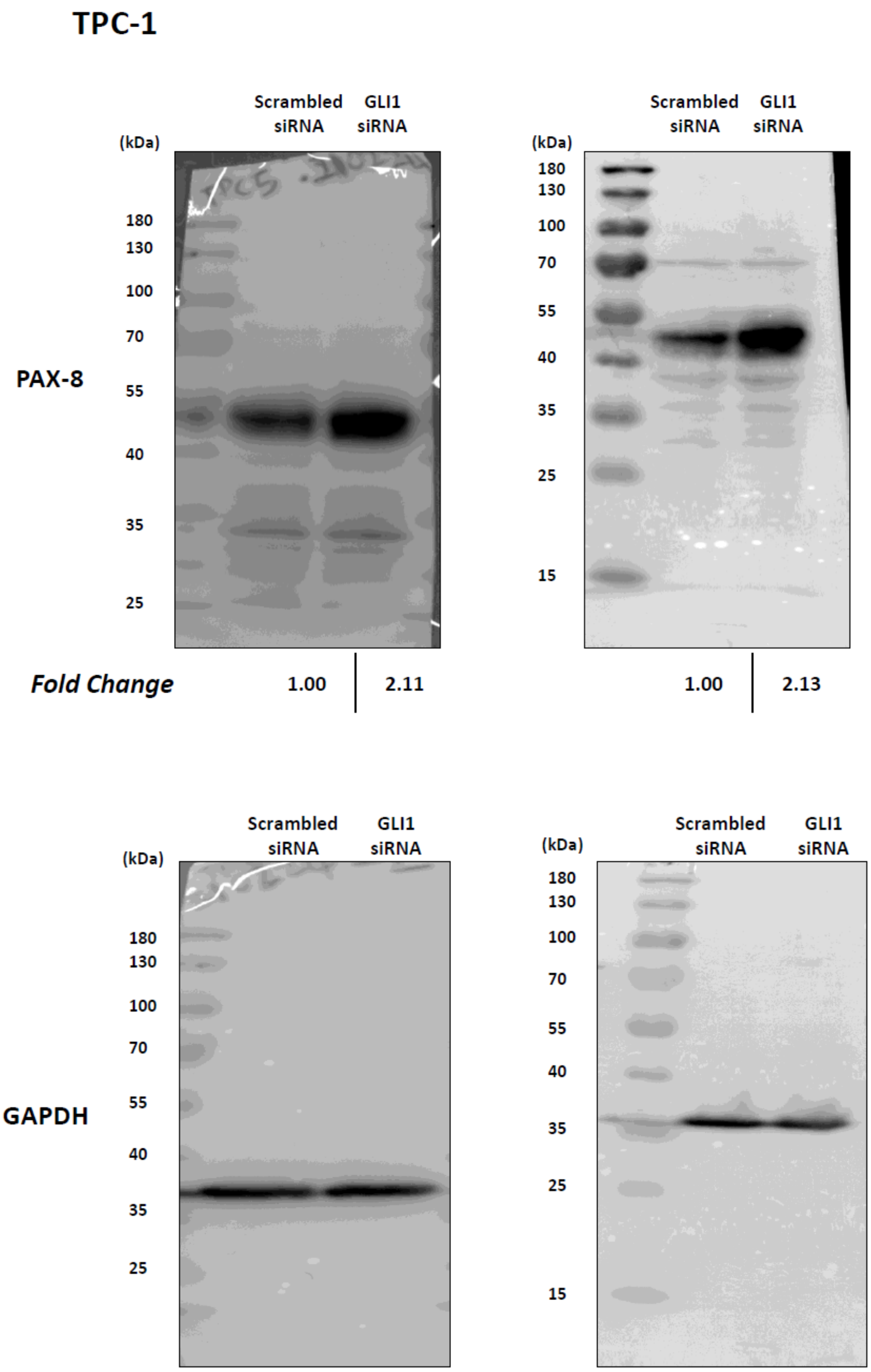
TPC-1
Scrambled siRNA
GLI1 siRNA
(kDa)
180
130
100
70
55
40
35
25
15
PAX-8
GAPDH
Fold Change
1.00
2.11
1.00
2.13

TPC-1

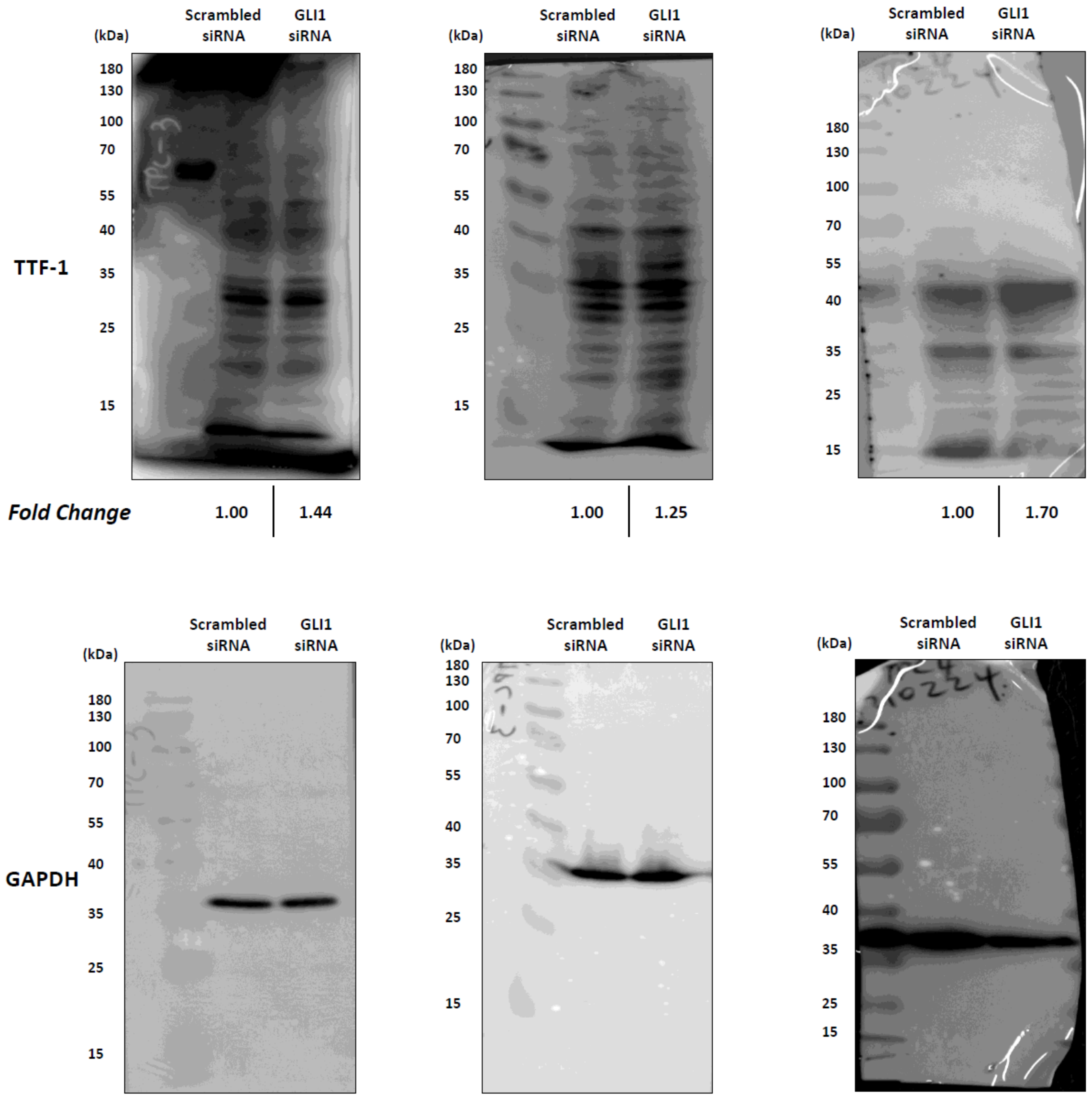

(kDa)
Scrambled siRNA
GLI1 siRNA
180
130
100
70
55
40
35
25
15
TTF-1
Fold Change
1.00
1.44
1.00
1.25
1.00
1.70
GAPDH

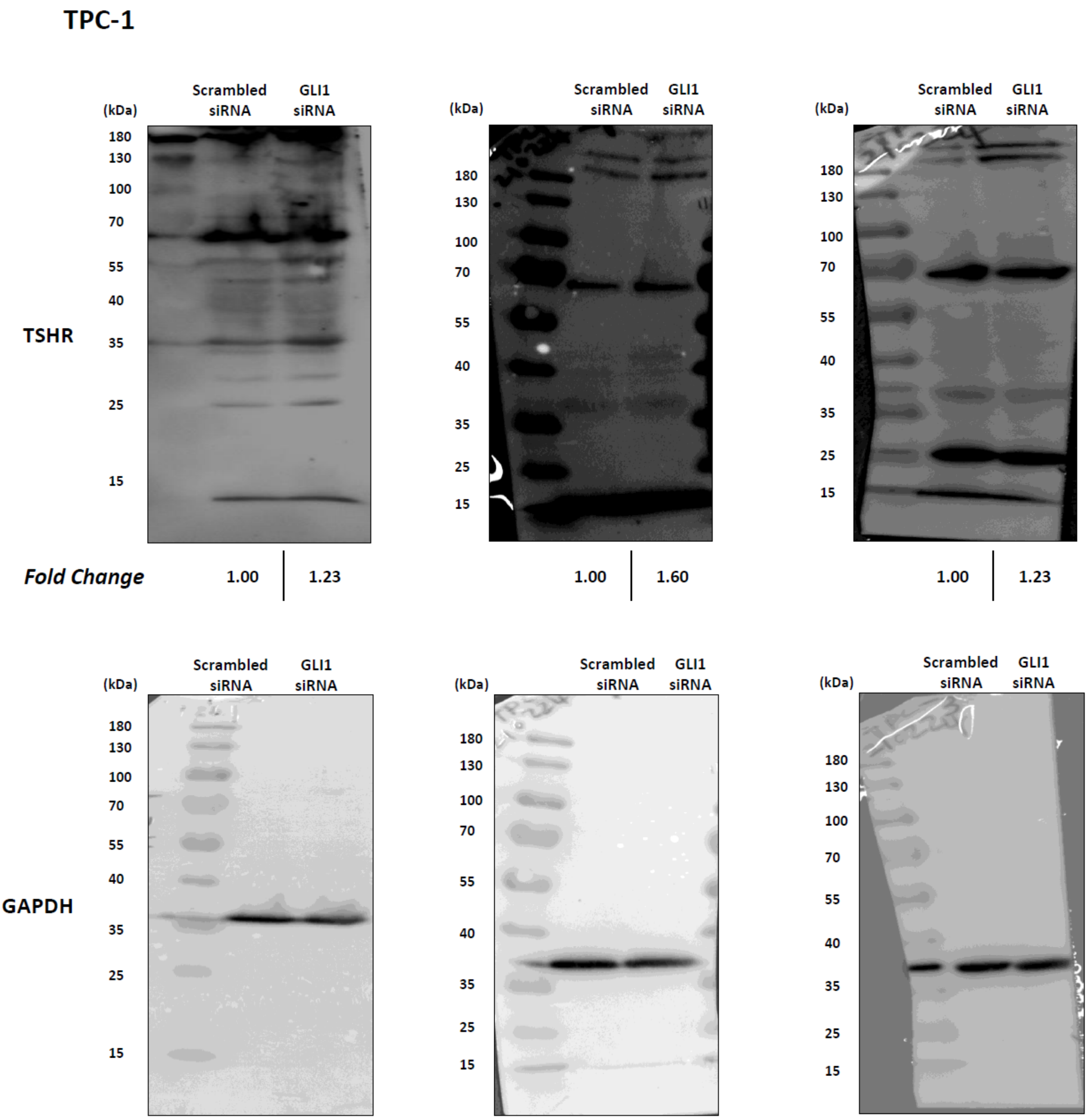
TPC-1
TSHR
Scrambled siRNA
GLI1 siRNA
(kDa)
180
130
100
70
55
40
35
25
15
Fold Change
1.00
1.23
1.00
1.60
1.00
1.23
GAPDH

TPC-1

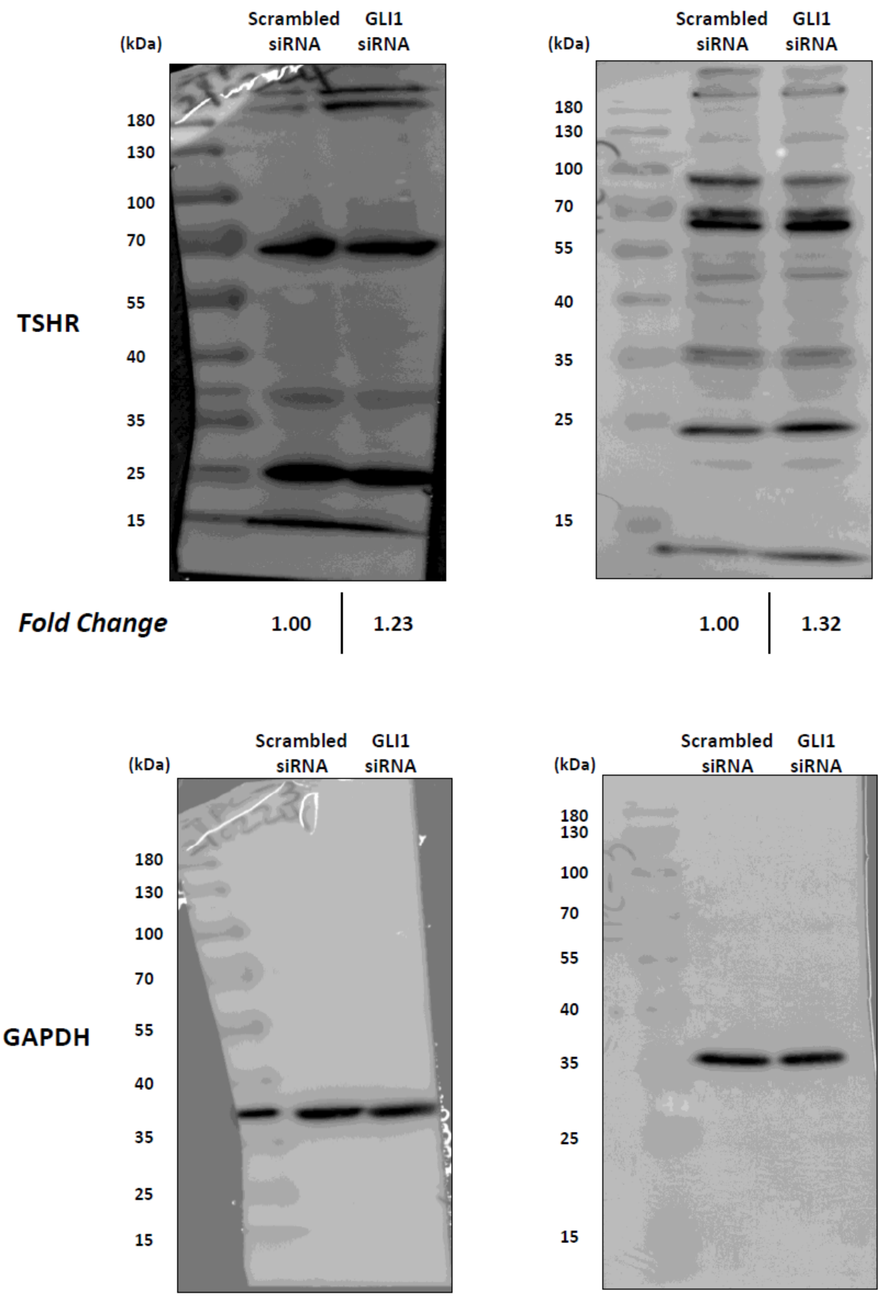

Figure S12. Raw data of Figure 3B.

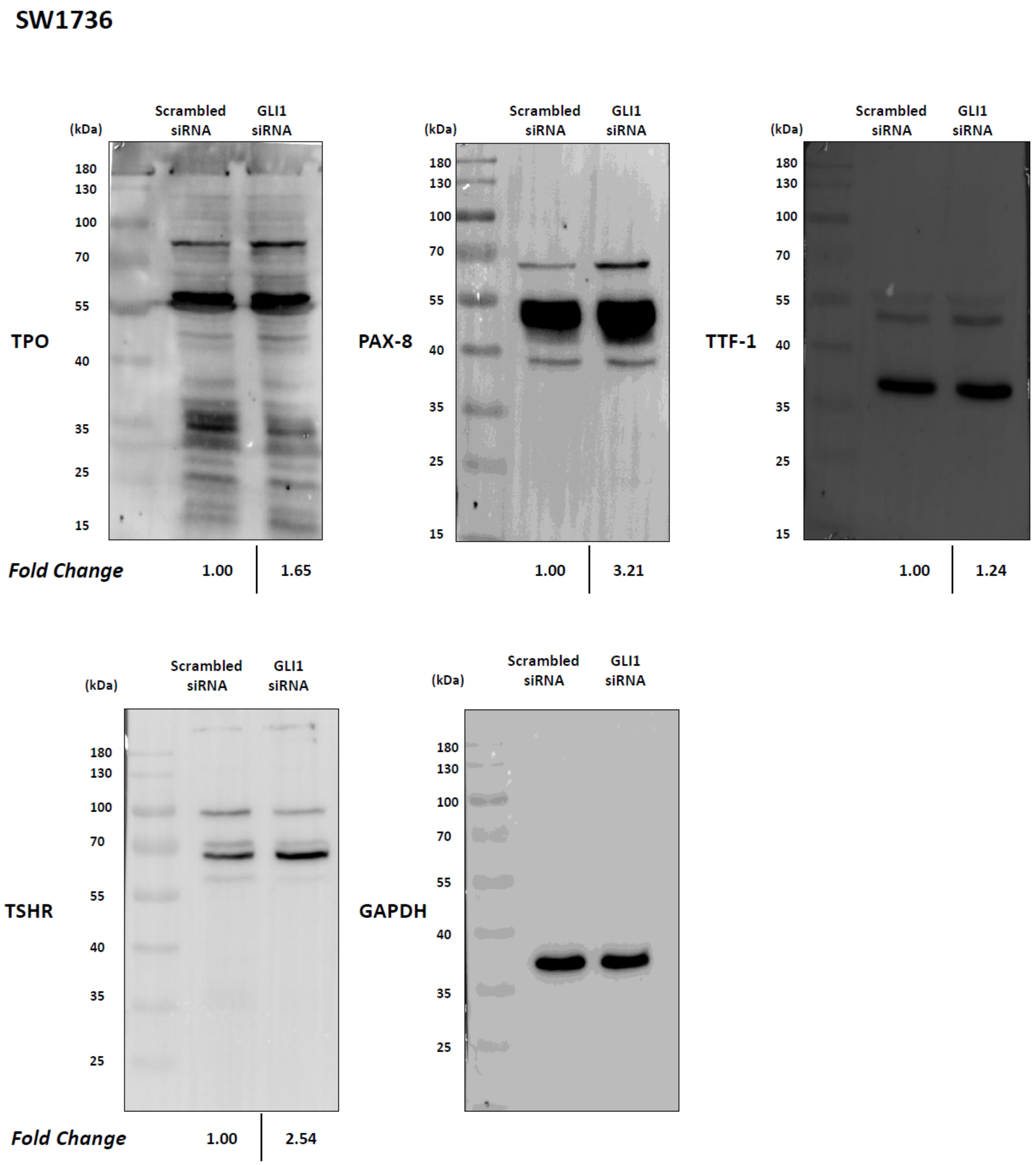

Figure S13. Raw data of Figure 3C.

SW1736

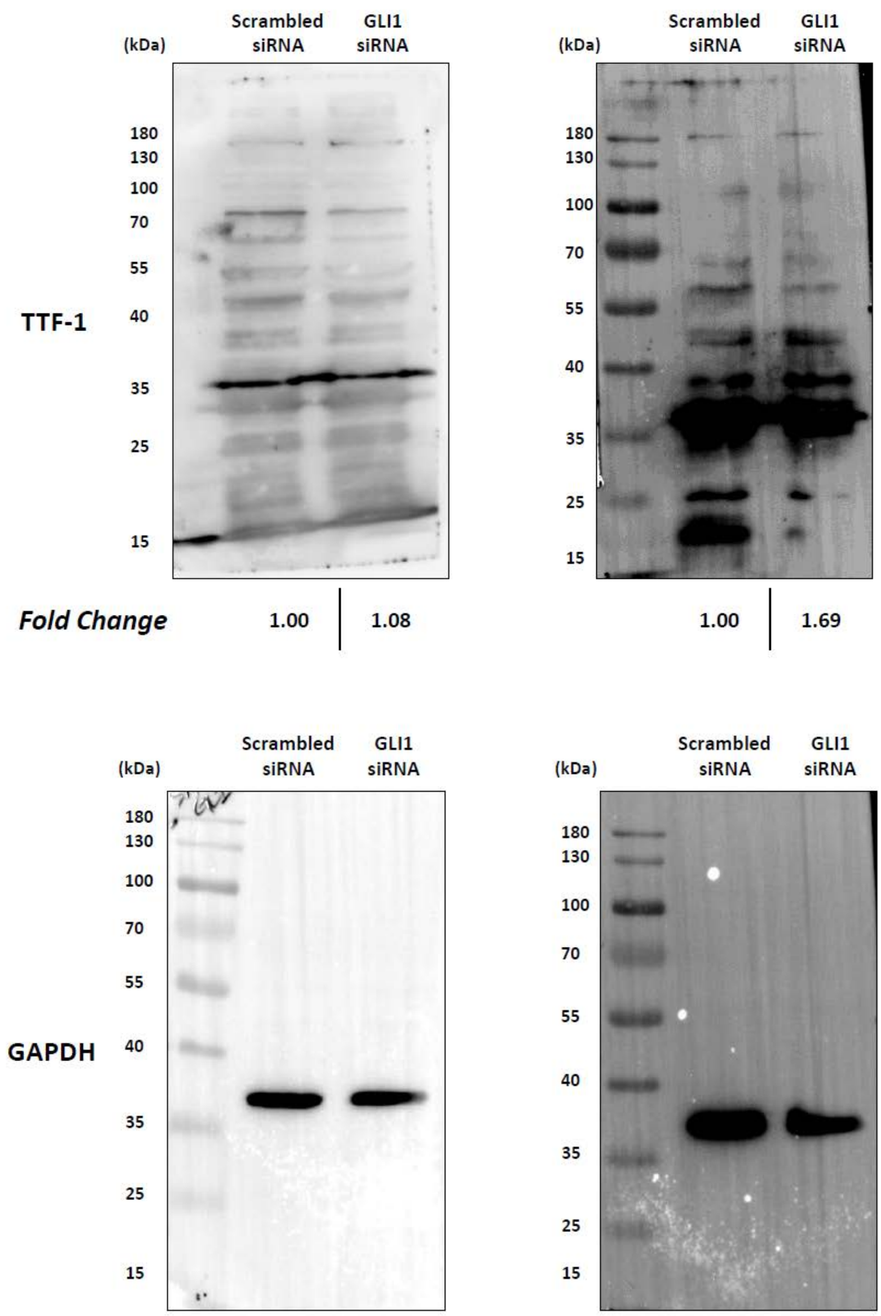

Scrambled siRNA
GLI1 siRNA
(kDa)
180
130
100
70
55
40
35
25
15
TTF-1
Scrambled siRNA
GLI1 siRNA
(kDa)
180
130
100
70
55
40
35
25
15
Fold Change
1.00
1.08
1.00
1.69
Scrambled siRNA
GLI1 siRNA
(kDa)
180
130
100
70
55
40
35
25
15
GAPDH
Scrambled siRNA
GLI1 siRNA
(kDa)
180
130
100
70
55
40
35
25
15

SW1736

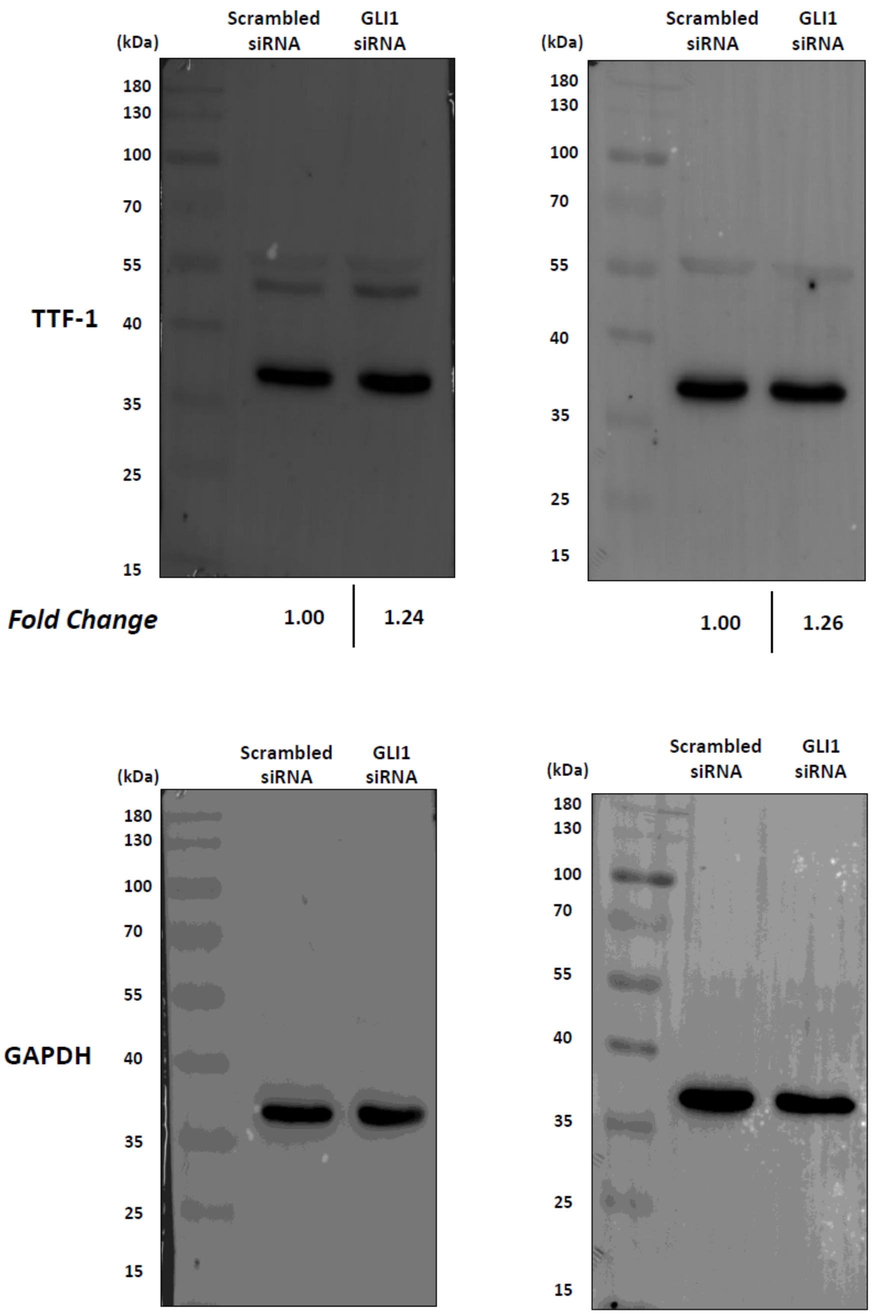

Scrambled siRNA
GLI1 siRNA
(kDa)
180
130
100
70
55
40
35
25
15
TTF-1
Fold Change
1.00
1.24
1.00
1.26
GAPDH

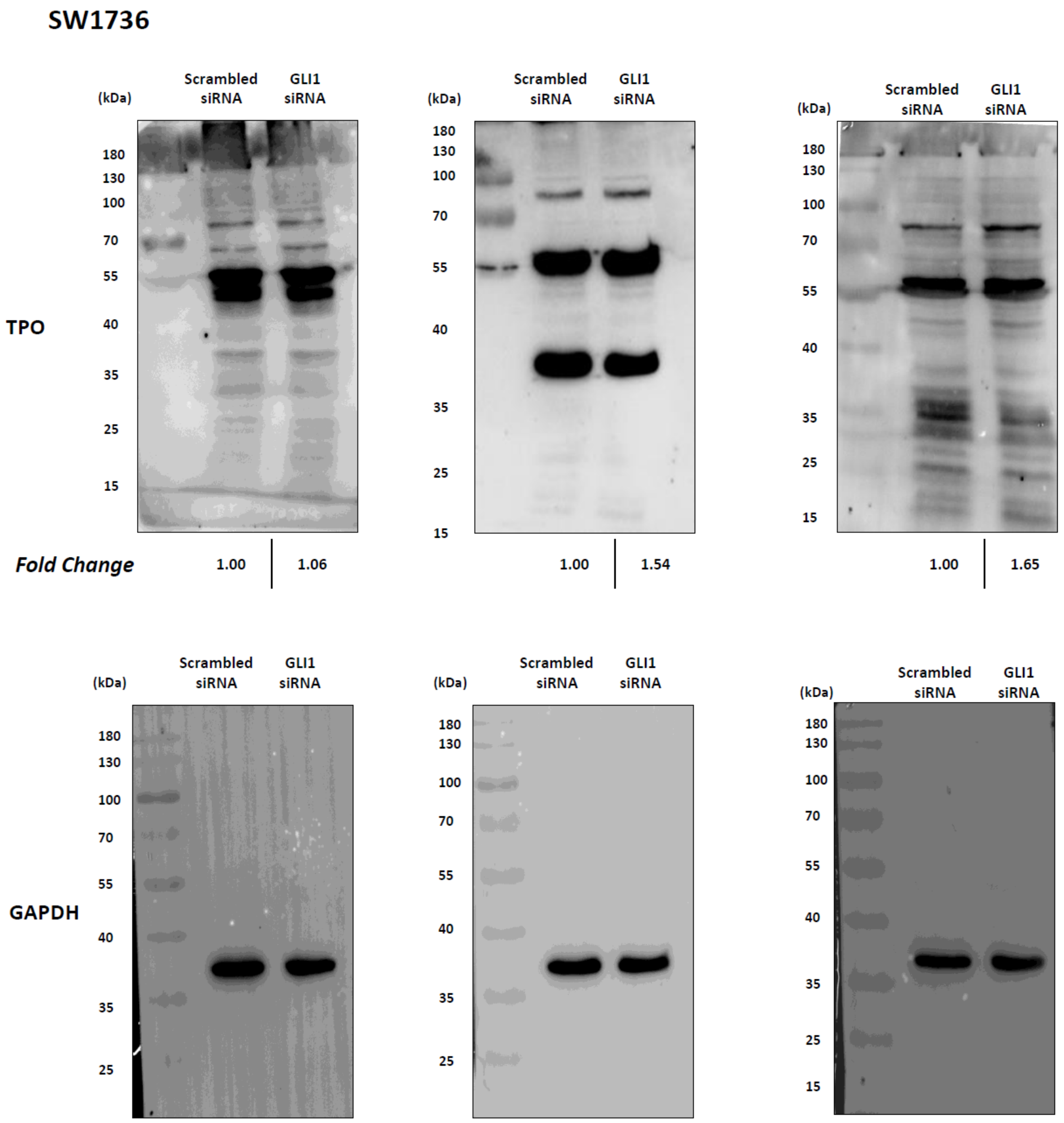
SW1736
(kDa)
Scrambled siRNA
GLI1 siRNA
180
130
100
70
55
40
35
25
15
TPO
Fold Change
1.00
1.06
1.00
1.54
1.00
1.65
GAPDH

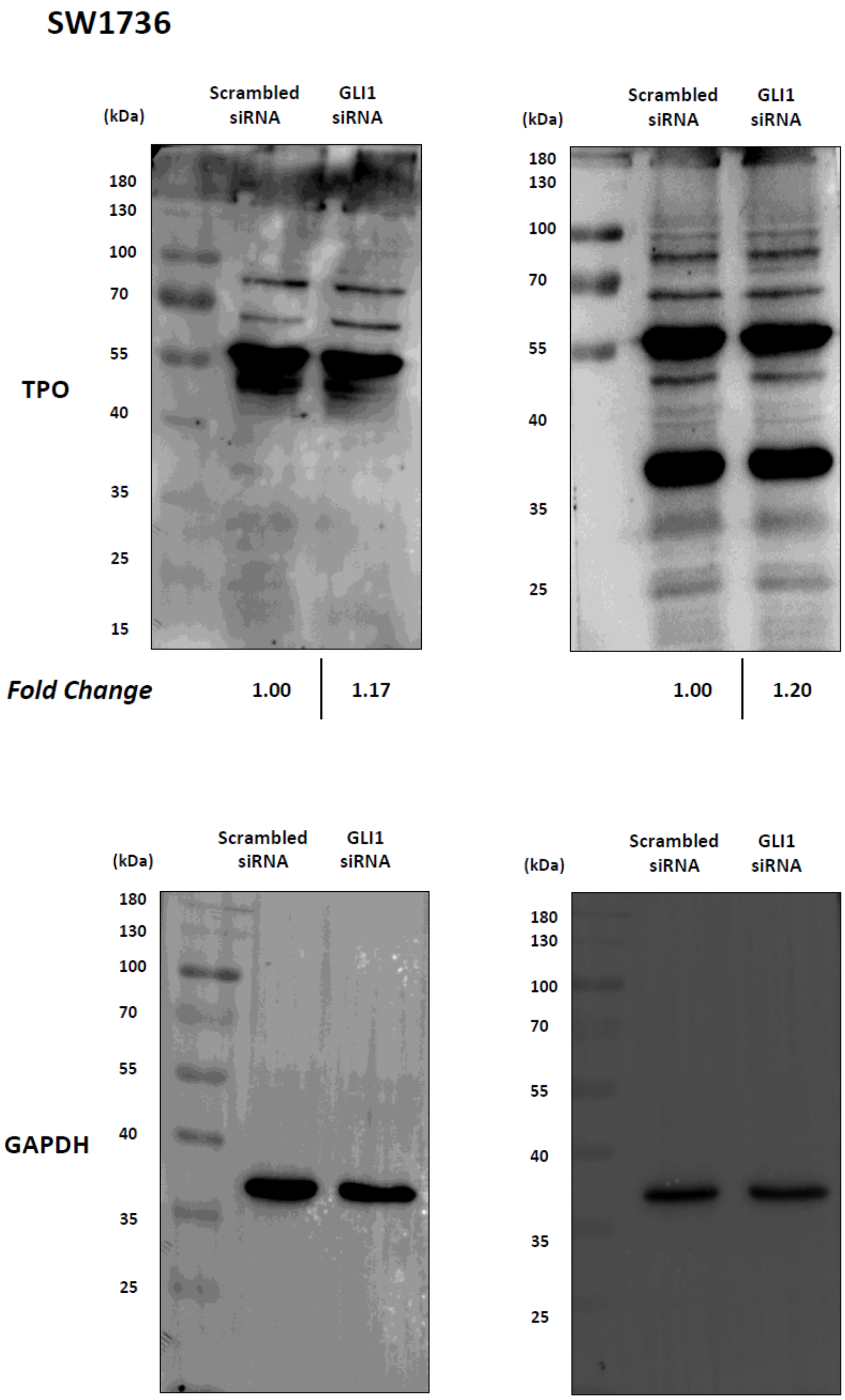
SW1736
(kDa)
Scrambled siRNA
GLI1 siRNA
180
130
100
70
55
40
35
25
15
TPO
Fold Change
1.00
1.17
1.20
GAPDH

SW1736

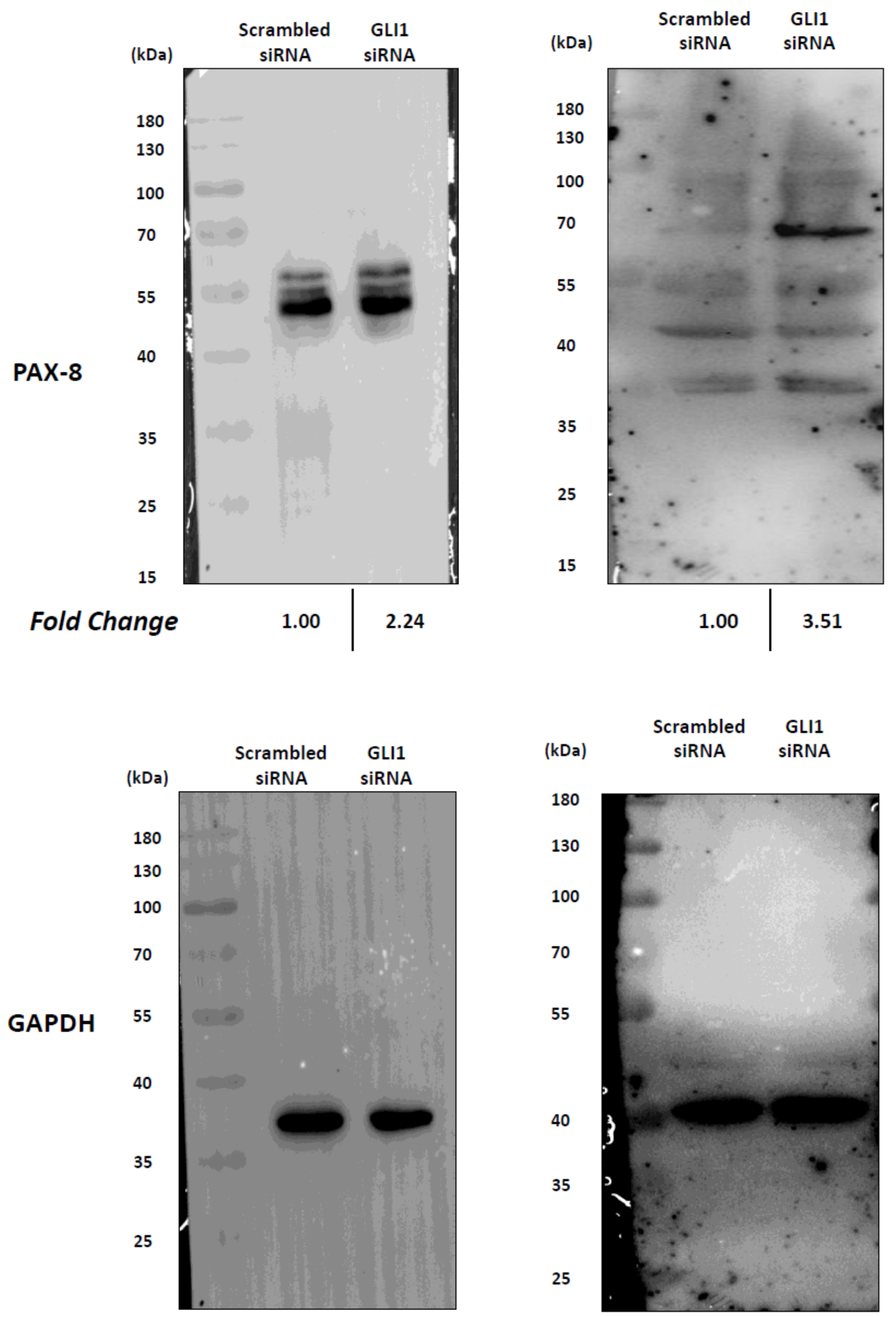
PAX-8
GAPDH
Scrambled siRNA
GLI1 siRNA
(kDa)
180
130
100
70
55
40
35
25
15
Fold Change
1.00
2.24
1.00
3.51

SW1736

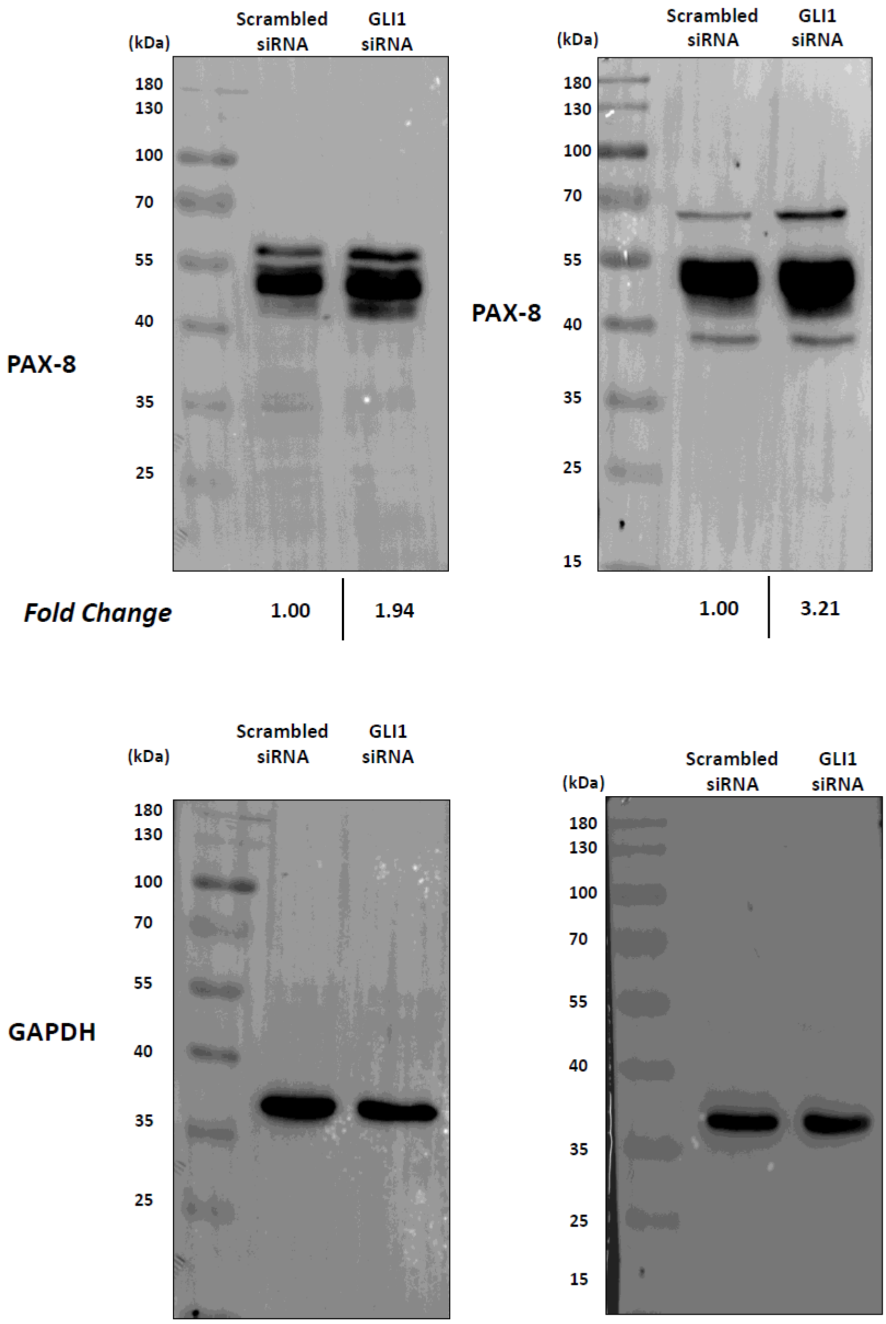
(kDa)
Scrambled siRNA
GLI1 siRNA
180
130
100
70
55
40
35
25
PAX-8
Fold Change
1.00
1.94
(kDa)
Scrambled siRNA
GLI1 siRNA
180
130
100
70
55
40
35
25
15
PAX-8
1.00
3.21
(kDa)
Scrambled siRNA
GLI1 siRNA
180
130
100
70
55
40
35
25
GAPDH
(kDa)
Scrambled siRNA
GLI1 siRNA
180
130
100
70
55
40
35
25
15

SW1736

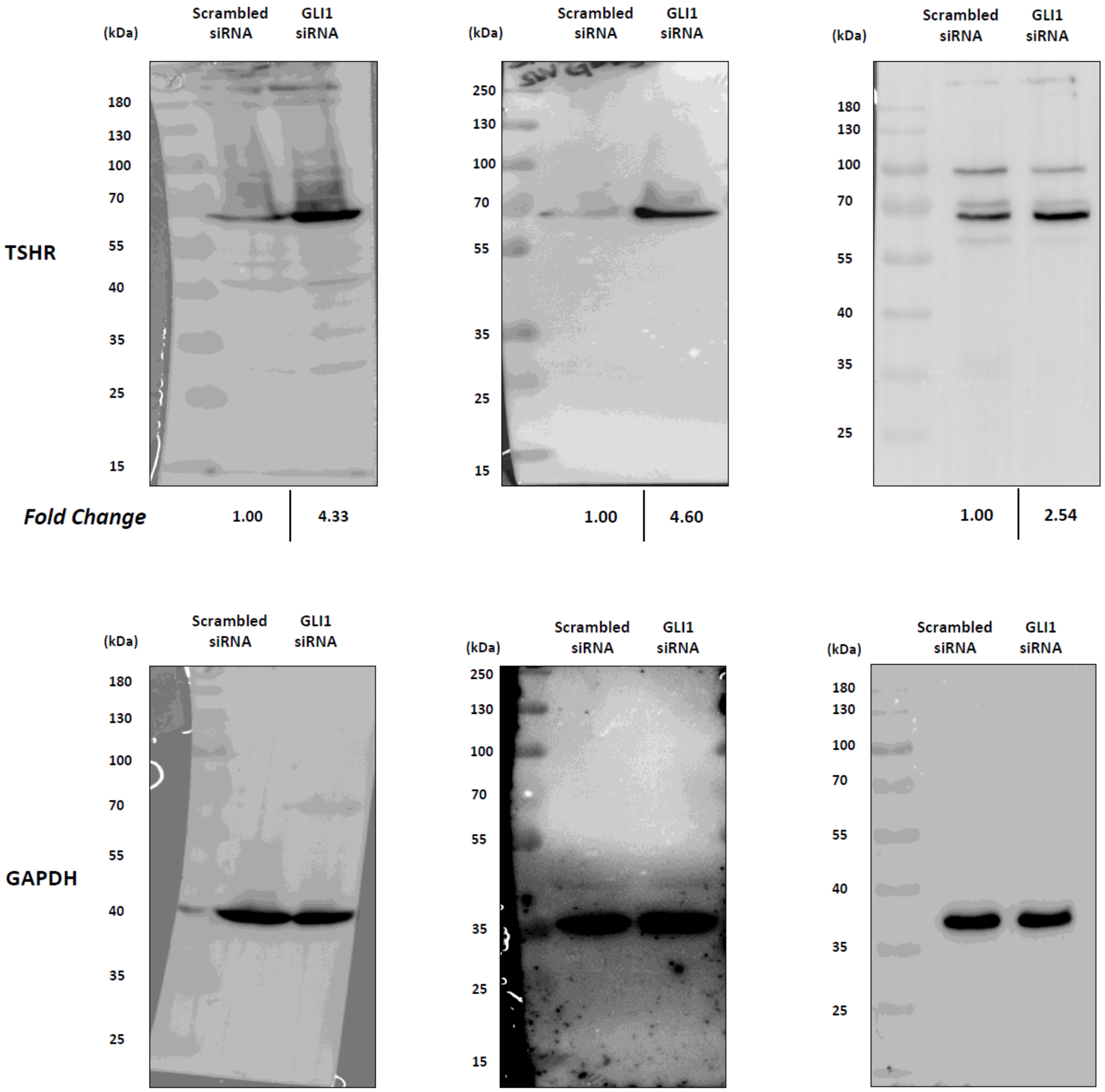

Figure S14. Raw data of Figure 3D.

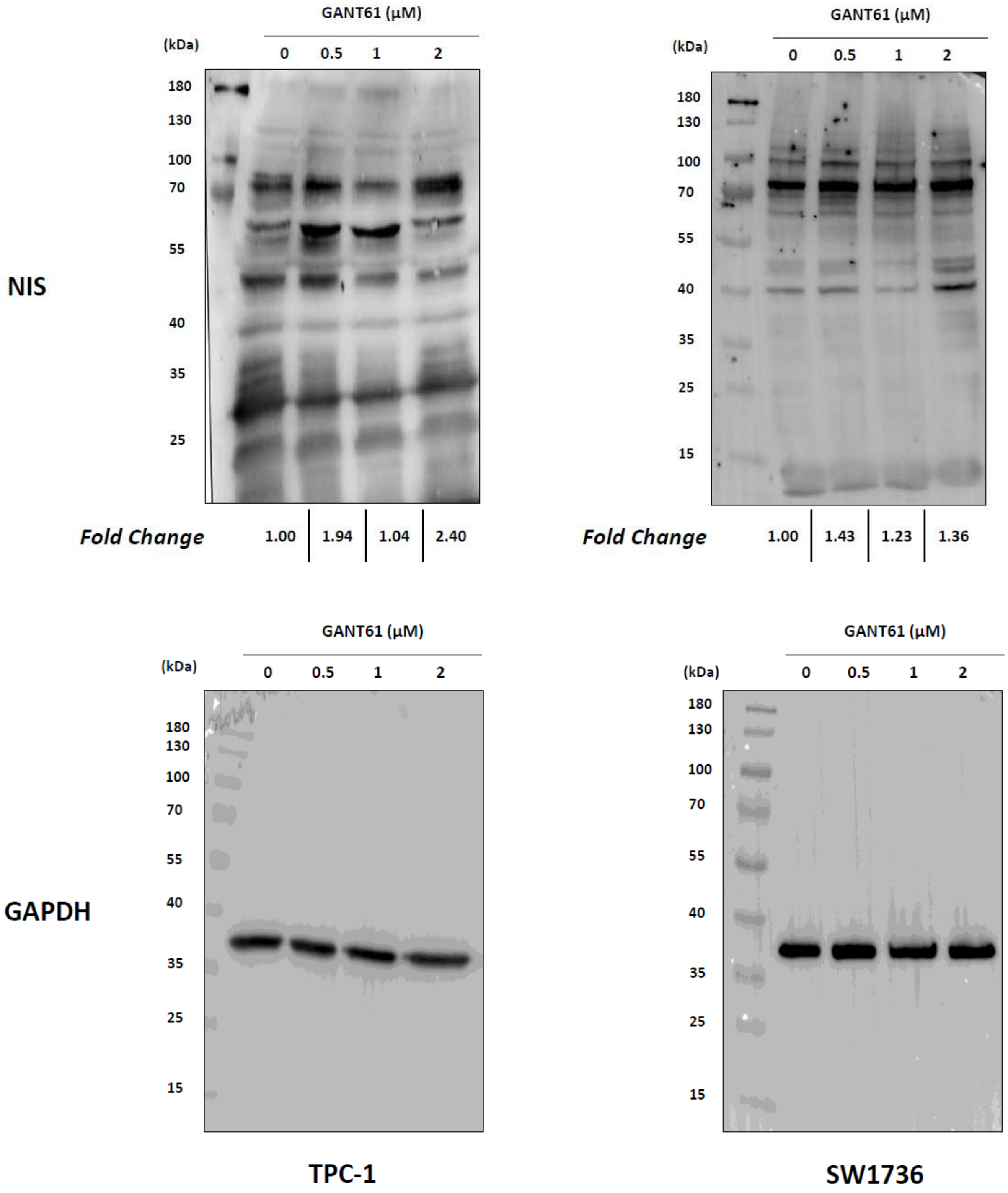
GANT61 (µM)
(kDa)
0 0.5 1 2
180
130
100
70
55
40
35
25
NIS
Fold Change 1.00 1.94 1.04 2.40
GANT61 (µM)
(kDa)
0 0.5 1 2
180
130
100
70
55
40
35
25
15
Fold Change 1.00 1.43 1.23 1.36
GANT61 (µM)
(kDa)
0 0.5 1 2
180
130
100
70
55
40
35
25
15
GAPDH
GANT61 (µM)
(kDa)
0 0.5 1 2
180
130
100
70
55
40
35
25
15
TPC-1
SW1736

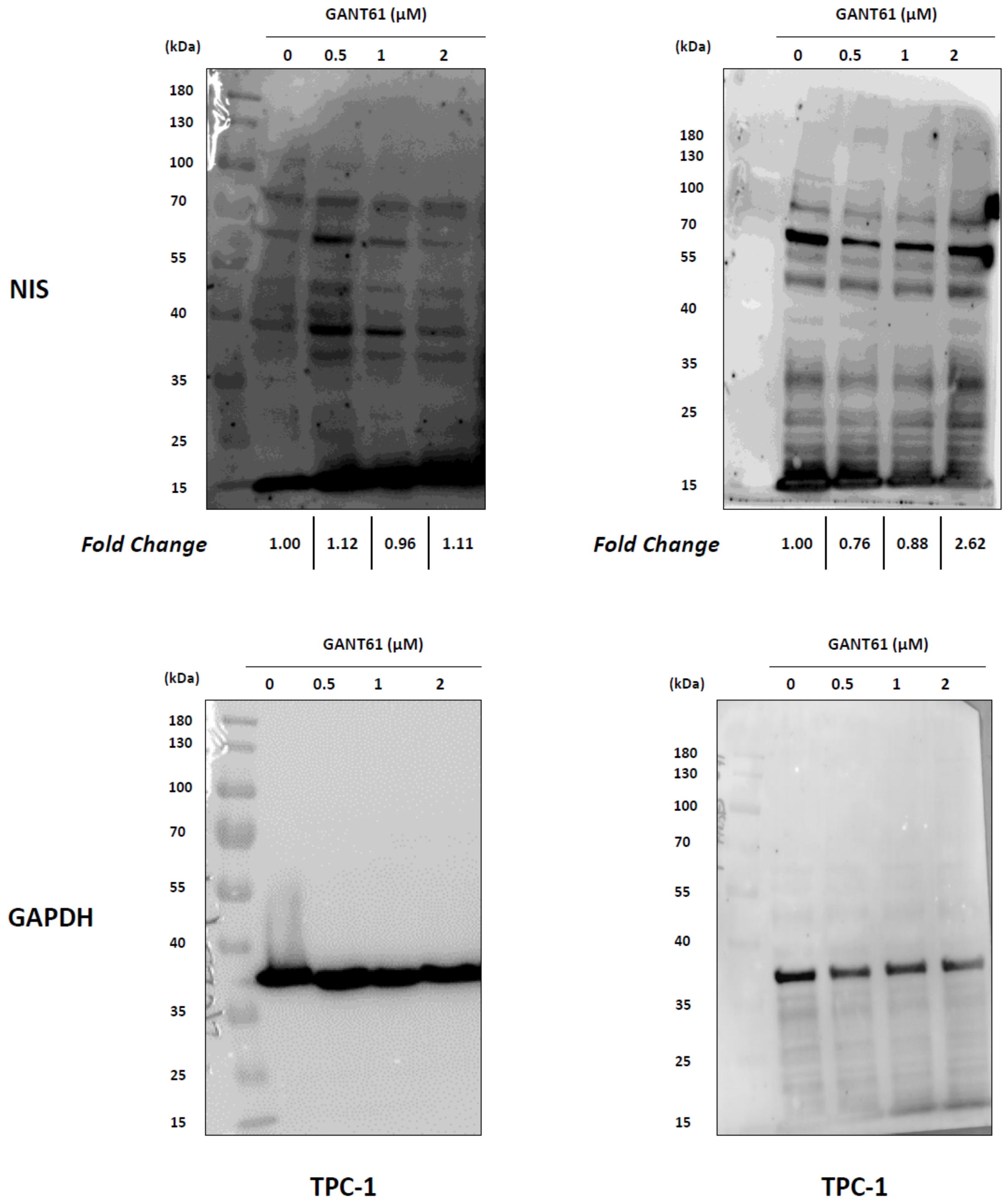

GANT61 (μM)
(kDa)
0
0.5
1
2
180
130
100
70
55
40
35
25
15
NIS
Fold Change
1.00
1.12
0.96
1.11
GANT61 (μM)
(kDa)
0
0.5
1
2
180
130
100
70
55
40
35
25
15
Fold Change
1.00
0.76
0.88
2.62
GANT61 (μM)
(kDa)
0
0.5
1
2
180
130
100
70
55
40
35
25
15
GAPDH
GANT61 (μM)
(kDa)
0
0.5
1
2
180
130
100
70
55
40
35
25
15
TPC-1
TPC-1

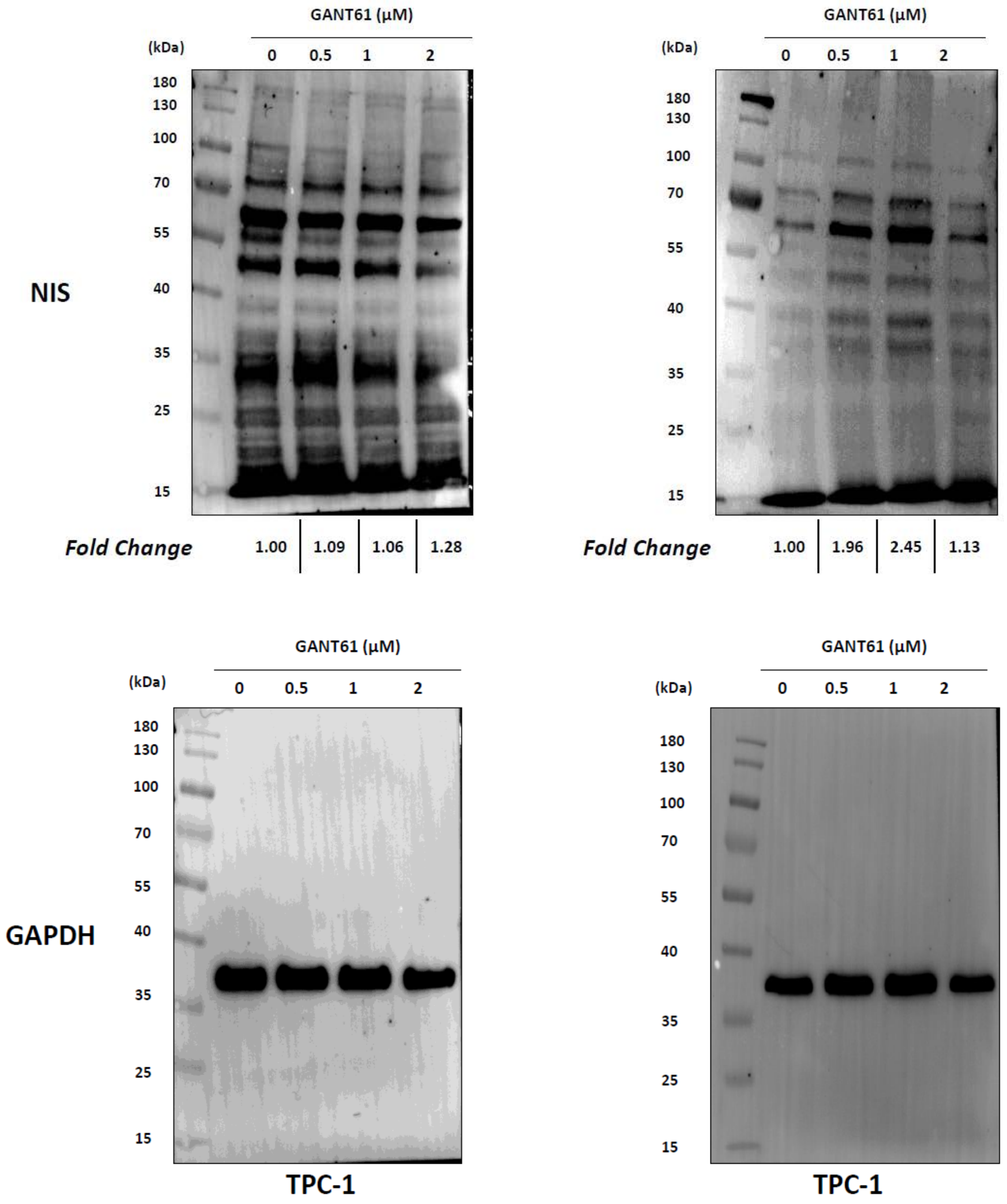
GANT61 (μM)
(kDa)
0 0.5 1 2
180
130
100
70
55
40
35
25
15
NIS
Fold Change 1.00 1.09 1.06 1.28
GANT61 (μM)
(kDa)
0 0.5 1 2
180
130
100
70
55
40
35
25
15
Fold Change 1.00 1.96 2.45 1.13
GANT61 (μM)
(kDa)
0 0.5 1 2
180
130
100
70
55
40
35
25
15
GAPDH
TPC-1
GANT61 (μM)
(kDa)
0 0.5 1 2
180
130
100
70
55
40
35
25
15
TPC-1

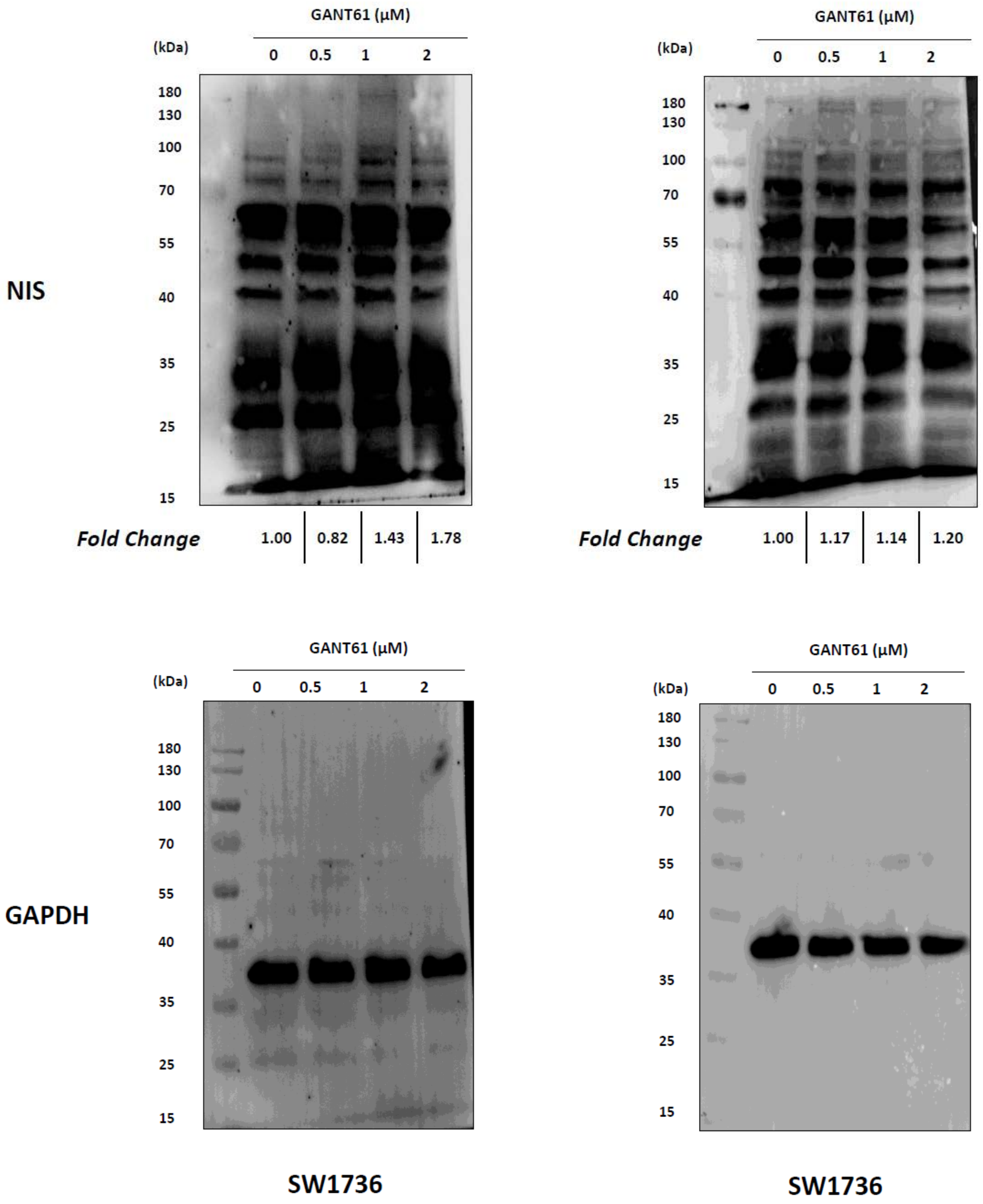
GANT61 (μM)
(kDa)
0 0.5 1 2
180
130
100
70
55
40
35
25
15
NIS
Fold Change
1.00 0.82 1.43 1.78
GANT61 (μM)
(kDa)
0 0.5 1 2
Fold Change
1.00 1.17 1.14 1.20
GAPDH
SW1736
SW1736

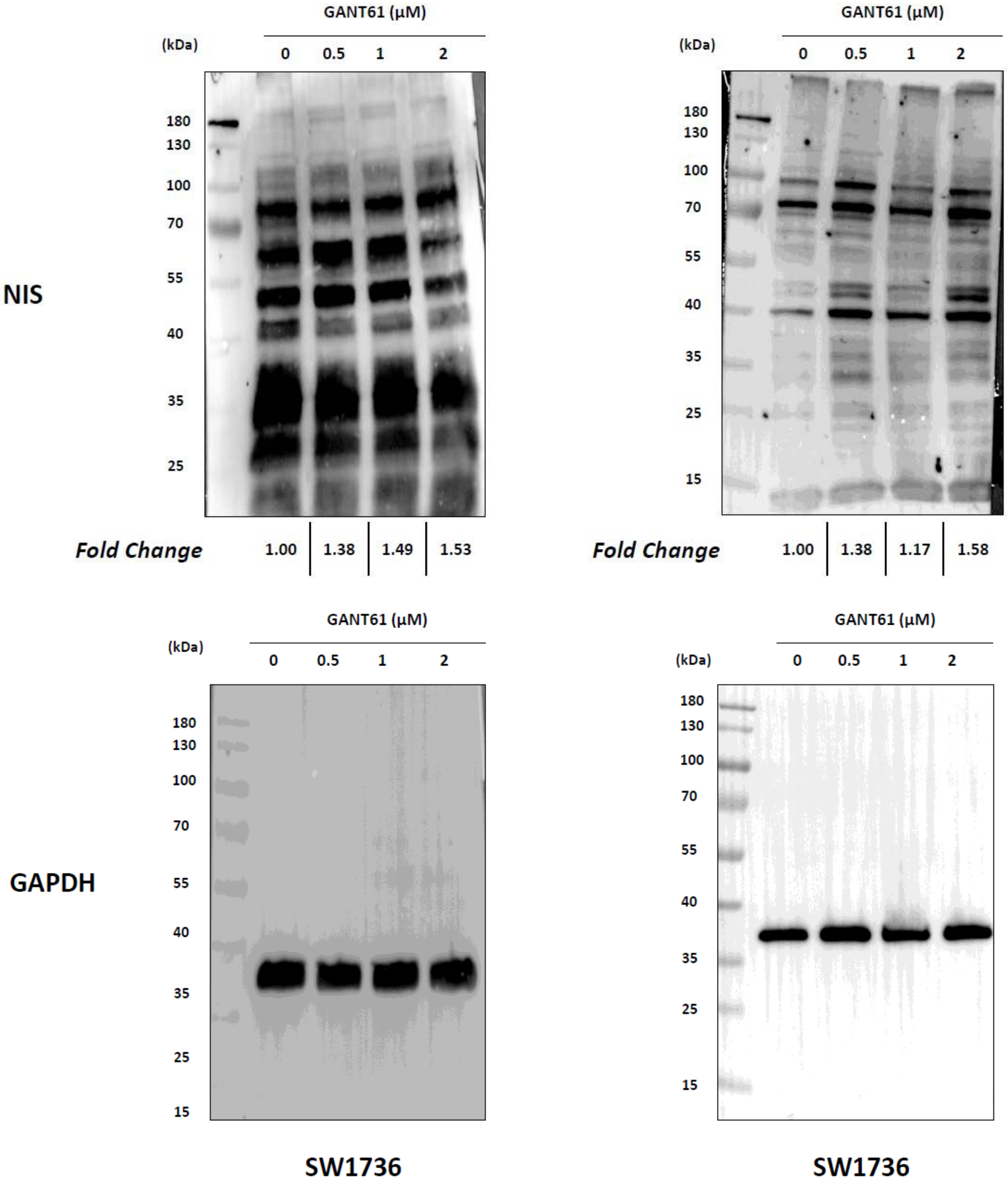

Figure S15. Raw data of Figure 5B.

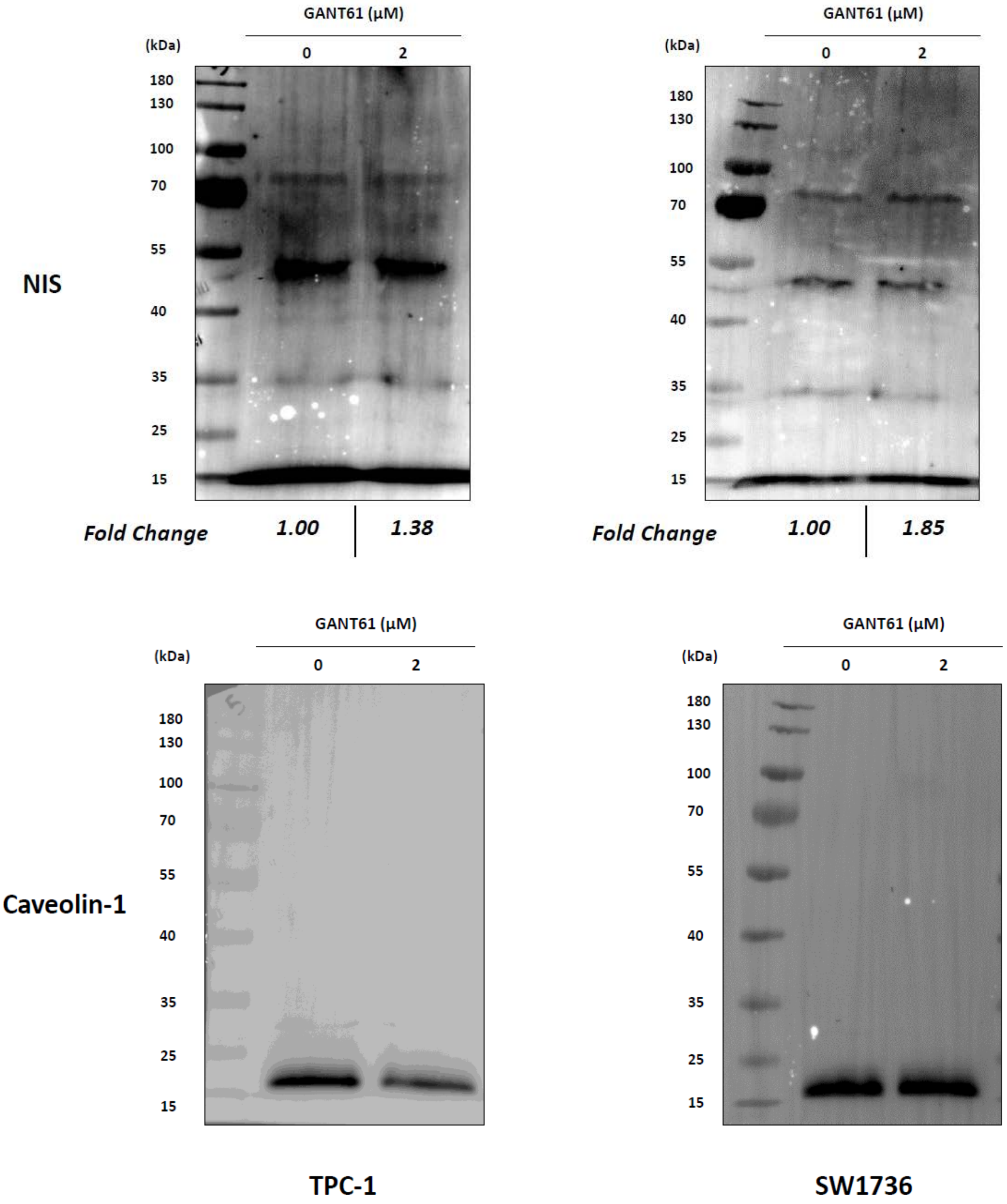

Figure S16: Raw data of Figure 5D.

Western blot analysis					
**Primary Antibodies**				**Secondary antibodies**	
**Name**	**Dilution ratio**	**Catalog Number**	**Manufacturer**	**Name**	**Dilution ratio**
**SHH**	**1:5000**	**#2207**	**Cell Signaling**	**Anti-Rabbit IgG, HRP-linked Antibody**	**1:10000**
**GLI1**	**1:4000**	**#3538**	**Cell Signaling**	**Anti-Rabbit IgG, HRP-linked Antibody**	**1:8000**
**SMO**	**1:4000**	**ab113438**	**Abcam**	**Anti-Rabbit IgG, HRP-linked Antibody**	**1:8000**
**PTCH1**	**1:4000**	**#2468**	**Cell Signaling**	**Anti-Rabbit IgG, HRP-linked Antibody**	**1:8000**
**NIS**	**1:4000**	**MS-1653**	**Thermo Fisher**	**Anti-Mouse IgG, HRP-linked Antibody**	**1:8000**
**PAX-8**	**1:1000**	**sc-81353**	**Santa Cruz**	**Anti-Mouse IgG, HRP-linked Antibody**	**1:2000**
**TTF-1**	**1:1000**	**sc-53136**	**Santa Cruz**	**Anti-Mouse IgG, HRP-linked Antibody**	**1:2000**
**Thyroperoxidase (TPO)**	**1:1000**	**sc-58432**	**Santa Cruz**	**Anti-Mouse IgG, HRP-linked Antibody**	**1:2000**
**TSH-receptor (TSHR)**	**1:1000**	**sc-515556**	**Santa Cruz**	**Anti-Mouse IgG, HRP-linked Antibody**	**1:2000**
**Caveolin-1**	**1:2500**	**#3267**	**Cell Signaling**	**Anti-Rabbit IgG, HRP-linked Antibody**	**1:5000**
**GAPDH**	**1:5000**	**sc-47724**	**Santa Cruz**	**Anti-Mouse IgG, HRP-linked Antibody**	**1:10000**

Scheme 1. List of used primary antibodies for western blot analysis.
